# Splicing variants in MYRF cause partial loss of function in the retinal pigment epithelium leading to nanophthalmos

**DOI:** 10.1172/jci.insight.194681

**Published:** 2026-02-26

**Authors:** Gabrielle M. Rozumek, Michelle L. Brinkmeier, Bin Guan, Su Qing Wang, Catherine Tower, Nina T. Yang, Rachel S. Lim, Dejuan Kong, Daniel Soden, Qitao Zhang, John Y.S. Han, Jason M.L. Miller, Lijin Dong, D. Ford Hannum, Sayoko E. Moroi, Julia E. Richards, Robert B. Hufnagel, Lev Prasov

**Affiliations:** 1 Department of Ophthalmology and Visual Sciences,; 2 Department of Pathology, and; 3 Department of Human Genetics, University of Michigan, Ann Arbor, Michigan, USA.; 4 National Eye Institute, NIH, Bethesda, Maryland, USA.; 5 Cellular and Molecular Biology Program, University of Michigan, Ann Arbor, Michigan, USA.; 6 Department of Ophthalmology and Visual Sciences, The Ohio State University, Columbus, Ohio, USA.; 7 Department of Epidemiology, The University of Michigan, Ann Arbor, Michigan, USA.; 8 Center for Integrated Healthcare Research, Kaiser Permanente Hawaii, Honolulu, Hawaii, USA.

**Keywords:** Genetics, Ophthalmology, Cytoskeleton, Embryonic development, Molecular genetics

## Abstract

Improper light focus on the retina, refractive error, is primarily caused by eye size differences and is the leading cause of vision loss worldwide. C-terminal variants in the Myelin regulatory factor (*MYRF*) gene, a retinal pigment epithelium–derived (RPE-derived) transcription factor, lead to isolated nanophthalmos characterized by a small, though structurally sound eye. However, other MYRF loss-of-function variants cause syndromic disease. To address this discrepancy, in vitro and animal studies were performed on a pathogenic C-terminal variant dG-MYRF (p.Gly1126fs30*, c.3376-1G>A). Human RPE cells or primary RPE transduced with dG-MYRF showed reduced target gene expression, with decreased steady-state levels of the C-terminal cleavage product, but normal cleavage and localization. A homozygous humanized MYRF C-terminal mouse model (*Myrf^humdG/humdG^*) was embryonic lethal by E18.5, while WT (*Myrf^humWT/humWT^*) mice were viable. Single-cell RNA-seq from E17.5 *Myrf^humdG/humdG^* and KO *RxCre;Myrf^fl/fl^* (E15.5 and P0) mice revealed shared differentially expressed genes, with decreased effect size in the *Myrf^humdG/humdG^* eyes. These findings support dG-MYRF as a hypomorphic allele. Additionally, 2 *MYRF* splicing variants creating nonfunctional isoforms were found in families with isolated nanophthalmos. Overall, hypomorphic *MYRF* alleles underlie isolated nanophthalmos, supporting a tissue-specific threshold effect and highlighting unique roles for the MYRF C-terminus in the RPE.

## Abstract 2

Dominant variants in MYRF partially altering gene function or dosage may lead to ocular disease only while total loss-of-function may lead to syndromic disease.

## Graphical abstract

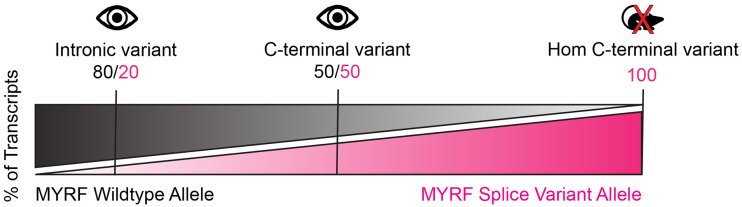

## Introduction

Myelin regulatory factor (MYRF) is a membrane-associated transcription factor initially identified for its role in oligodendrocyte maturation and myelination (1). It is a type II transmembrane protein that homotrimerizes in the endoplasmic reticulum (ER) lumen and autocleaves to release an N-terminal fragment that translocates to the nucleus and acts as a transcriptional activator (2). More recently, pathogenic variants in *MYRF* have been associated with an ocular-cardiac-urogenital syndrome featuring congenital heart defects, diaphragmatic hernias, pulmonary hypoplasia, genital abnormalities, and high hyperopia (3–7). *MYRF* variants have also been described in isolated nanophthalmos, an ocular disease featuring a small but structurally normal eye, with resulting extreme farsightedness (hyperopia). C-terminal frameshift variants have been identified in multiple large pedigrees with predominantly isolated nanophthalmos, suggesting a unique role for the MYRF C-terminus in the eye (8, 9). However, it is not clear why some variants in *MYRF* result in isolated ocular disease while others produce syndromic phenotypes.

In the eye, MYRF is predominantly and highly expressed in the developing and mature retinal pigment epithelium (RPE) (8, 10). Conditional loss of mouse *Myrf* in the eye leads to defects in RPE development and retinal degeneration in mice, with perturbations in TGF-β/BMP signaling, pigmentation, cell structure, and cell viability (10). Although the mouse models of *Myrf* show disparate phenotypes compared with patients, they are invaluable for understanding retinal disease pathogenesis in vivo (8, 11, 12).

**Figure 1:**
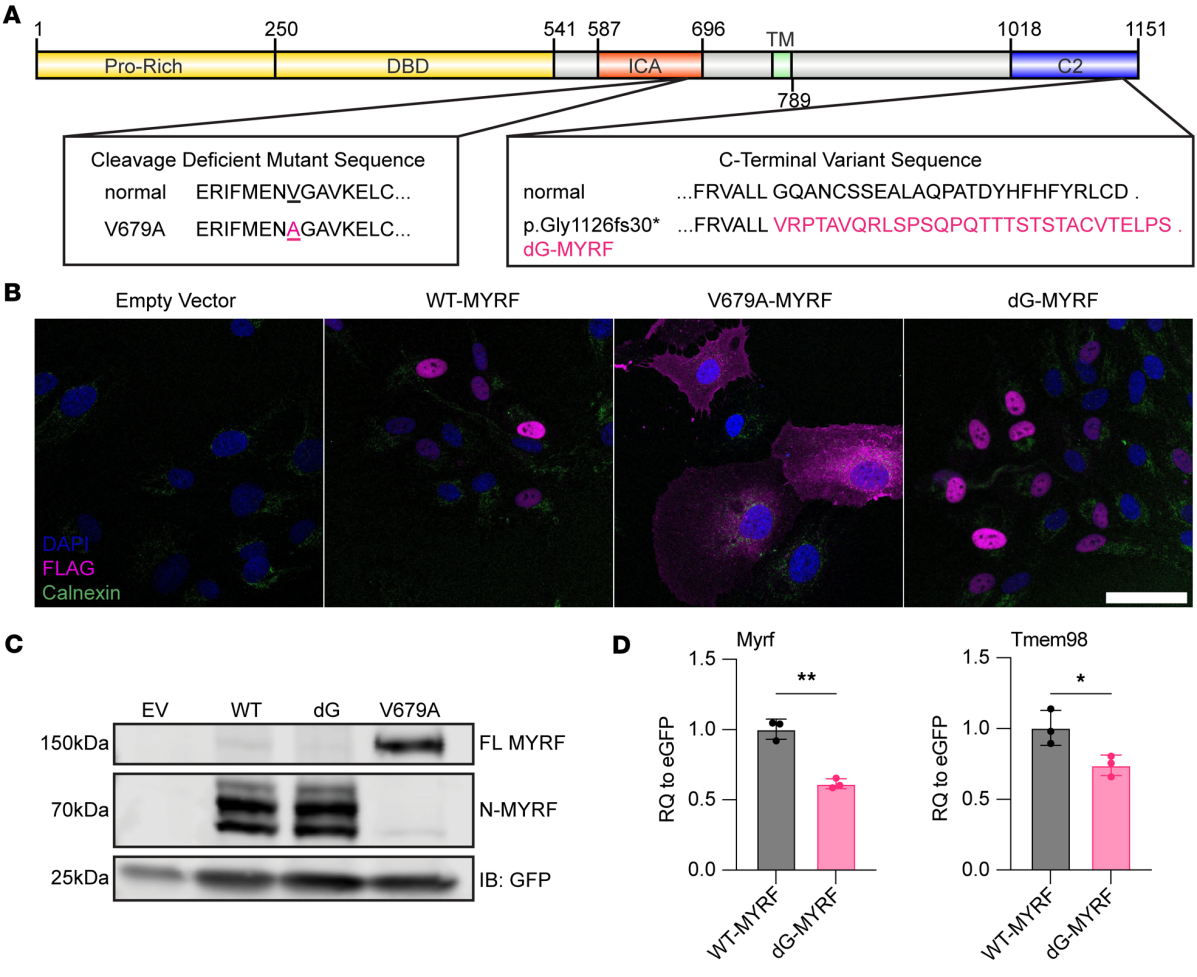
Processing of C-terminal MYRF variant in vitro. (**A**) Diagram of amino acid changes in WT-MYRF, dG-MYRF, and cleavage deficient variant V679A-MYRF. ProRich, proline rich; DBD, DNA binding domain; ICA, intramolecular chaperone auto-processing; TM, transmembrane; C2, C-terminal. (**B**) Localization of FLAG-tagged MYRF constructs in ARPE-19 cells show normal nuclear localization of dG-MYRF (*n* = 3). Scale bar: 50 μm. (**C**) Western blot of transfected ARPE-19 cells shows no change in cleavage of N-terminal fragment (*n* = 3). (**D**) qPCR analysis of RNA from ARPE-19 cells transduced with dG-MYRF, compared with WT-MYRF show decreased levels of transcripts for total *Myr*f and endogenous *Tmem9*8 mRNA (*n* = 3) by Student’s *t* test. *P < 0.05, **P < 0.01.

Two large nanophthalmos families have been reported with genetic changes that lead to the same C-terminal frameshift mutation in *MYRF* causing a 31–amino acid extension in the last exon (8, 9). Interestingly, a cluster of patients with pathogenic variants located in the conserved C2 domain manifest with predominantly ocular diseases (Figure 1) (13). While the functional consequences of variants in the DNA binding domain (DBD) and intramolecular chaperone autocleavage (ICA) domain have been well studied (14, 15), there has been little to no progress on understanding the function or importance of the ER-resident, conserved C2 domain.

Toward that end, we generated a humanized mouse model of the C-terminal *Myrf* variant and performed in vitro functional assays in RPE cell lines and primary human RPE cultures to better understand the molecular mechanism by which C-terminal extension alleles cause disease. Furthermore, we identified 2 additional rare splice site variants in *MYRF* in families with isolated nanophthalmos, supporting this as a more common mechanism for disease pathogenesis. Together, our studies support that partial loss-of-function alleles in *MYRF* contribute to isolated nanophthalmos and provide mechanistic insights into the role of the MYRF C2 domain.

## Results

### C-terminal MYRF variant is processed normally but shows decreased stability and transcriptional activity.

C-terminal *MYRF* variants and splicing variants are associated with predominantly ocular phenotypes and have familial inheritance, while early truncating and ICA and DBD variants are associated with syndromic phenotypes and predominantly occur de novo (9, 13, 15–17). Given this discrepancy, the C-terminal frameshift alleles could act as dominant negative or hypomorphic alleles. To distinguish these models, we evaluated localization, cleavage, and protein stability of the C-terminal variant in vitro in the ARPE-19 human RPE-like cells. When overexpressed with lentiviral vectors, both WT MYRF and C-terminal frameshift MYRF (dG-MYRF) (p.Gly1126fs30*) showed nuclear localization, while a known cleavage-deficient mutant V679A-MYRF (14) displayed only cytoplasmic localization and was excluded from the nucleus (Figure 1, A and B). Since homotrimerization at the ER lumen is necessary for cleavage of the N-terminal MYRF fragment and its subsequent translocation to the nucleus (1, 2, 18, 19), our results support a model in which dG-MYRF can homotrimerize and cleave.

To further evaluate this, we used Western blotting to systematically define the cleavage dynamics (ratio of full length/cleaved) of the dG-MYRF variant compared with WT-MYRF in ARPE-19 cells. We observed faint bands for full-length FLAG-MYRF (160 kDa) in both WT-MYRF and dG-MYRF and strong transcriptionally active N-MYRF cleavage products (~70 kDa). In contrast, the cleavage-deficient mutant, V679A-MYRF, shows 1 strong full-length MYRF band (Figure 1C). Given normal cleavage and localization, we next evaluated protein stability through computational tools examining degree of disorder Predictor of Natural Disordered Regions (PONDR) and protein folding (AlphaFold2) (20–23). PONDR uses neural networks trained on ordered and disordered regions of short and/or long regions of amino acid sequences from NMR or x-ray crystallography data to predict intrinsic disordered regions. PONDR analysis predicted that dG-MYRF alters the C-terminus from a highly ordered to a highly disordered structure (Supplemental Figure 1A; supplemental material available online with this article; https://doi.org/10.1172/jci.insight.194681DS1). De novo structural modeling using AlphaFold2 predicted the loss of a β-sheet and alpha-helix structure in the conserved C2 domain of the C-terminal frameshift variant of *MYRF* (Supplemental Figure 1, B and C). To experimentally validate this, cycloheximide pulse-chase experiments were conducted in ARPE-19 cells transduced with WT-MYRF and dG-MYRF. Protein extracts were analyzed by Western blotting using antibodies to the N-terminus or C-terminus of MYRF. The rates of decay of the C-terminal and N-terminal MYRF cleavage product were unchanged in the dG-MYRF variant following a 24-hour chase (Supplemental Figure 2A). However, steady state levels of C-terminal cleavage product for dG-MYRF were reduced compared with WT (Supplemental Figure 2, B and C). This was unlikely due to reduced epitope detection because the antibody was raised to an epitope present in both forms of MYRF (anti-MYRF^393-766AA^) (1), and the C2 domain is predicted to fold independently.

To evaluate the functional effect of the dG-MYRF variant on transcriptional activity, we tested the ability of dG-MYRF to autoregulate *MYRF* transcription and its downstream target *TMEM98* using qPCR, as compared with an internal transduction GFP control, present in each of the constructs. ARPE-19 cells transduced with dG-MYRF showed decreased levels of endogenous *MYRF* and *TMEM98* mRNA expression (relative to GFP) as compared with WT-MYRF (Figure 1D).

**Figure 2:**
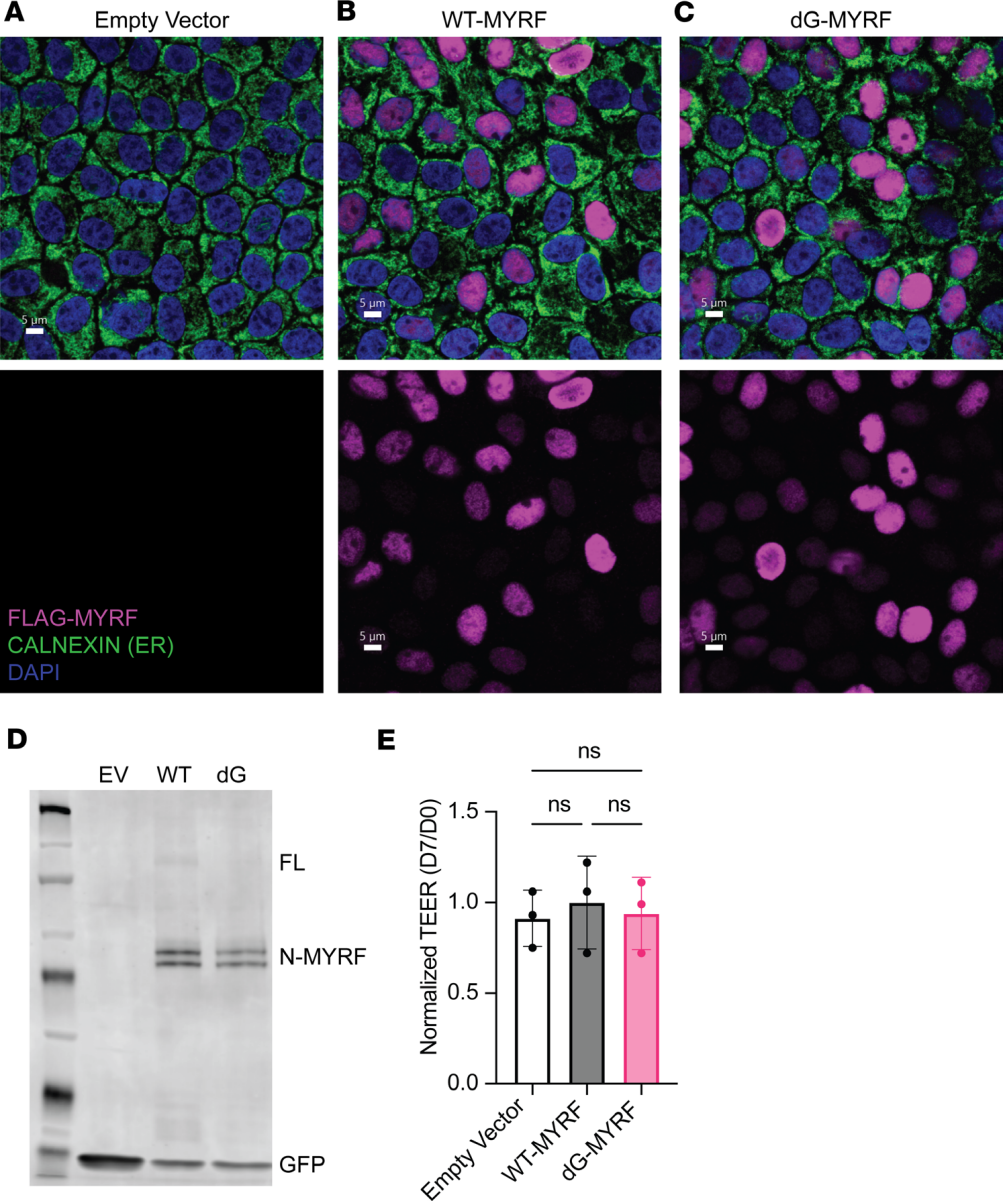
Processing and functional studies of C-terminal MYRF variant in human primary RPE. (**A**–**C**) Localization of FLAG-tagged MYRF constructs in mature human primary RPE show normal nuclear localization of dG-MYRF. Scale bar: 5 μm. (**D**) Western blot of transduced human primary RPE cells shows no change in cleavage of N-terminal fragment. (**E**) Functional assessment of barrier function via TEER (normalized to pretransduction TEER value for each replicate) shows no significant difference in TEER 1 week after transduction with Empty Vector, WT-MYRF, or dG-MYRF by 1-way ANOVA (all experiments are 3 reps from *n* = 2 donors).

To evaluate whether RPE differentiation state and polarization alter cleavage dynamics of MYRF, we repeated key ARPE-19 experiments in primary human RPE cultures grown on microporous supports, as described previously (24). We again observed normal localization and cleavage of virally transduced WT-MYRF and dG-MYRF primary RPE cultures (Figure 2, A–D). Additionally, transepithelial electrical resistance (TEER) was measured as a proxy for barrier function and overall cell health since we have previously demonstrated the close relationship between assays for cell toxicity and declines in TEER in primary RPE cultures (25). We observed no difference in TEER in primary RPE transduced with Empty Vector, WT-MYRF, or dG-MYRF 1 week after transduction compared with the same well before transduction (Figure 2E). Overall, the results obtained in the ARPE-19 cell line were consistent with results seen in mature and polarized human primary RPE cultures, demonstrating the dG-MYRF variant does not affect localization, processing, barrier function, or overall cell health but seems to reduce transcriptional activation function of MYRF.

### Mouse model of human C-terminal MYRF is prenatal lethal.

**Figure 3:**
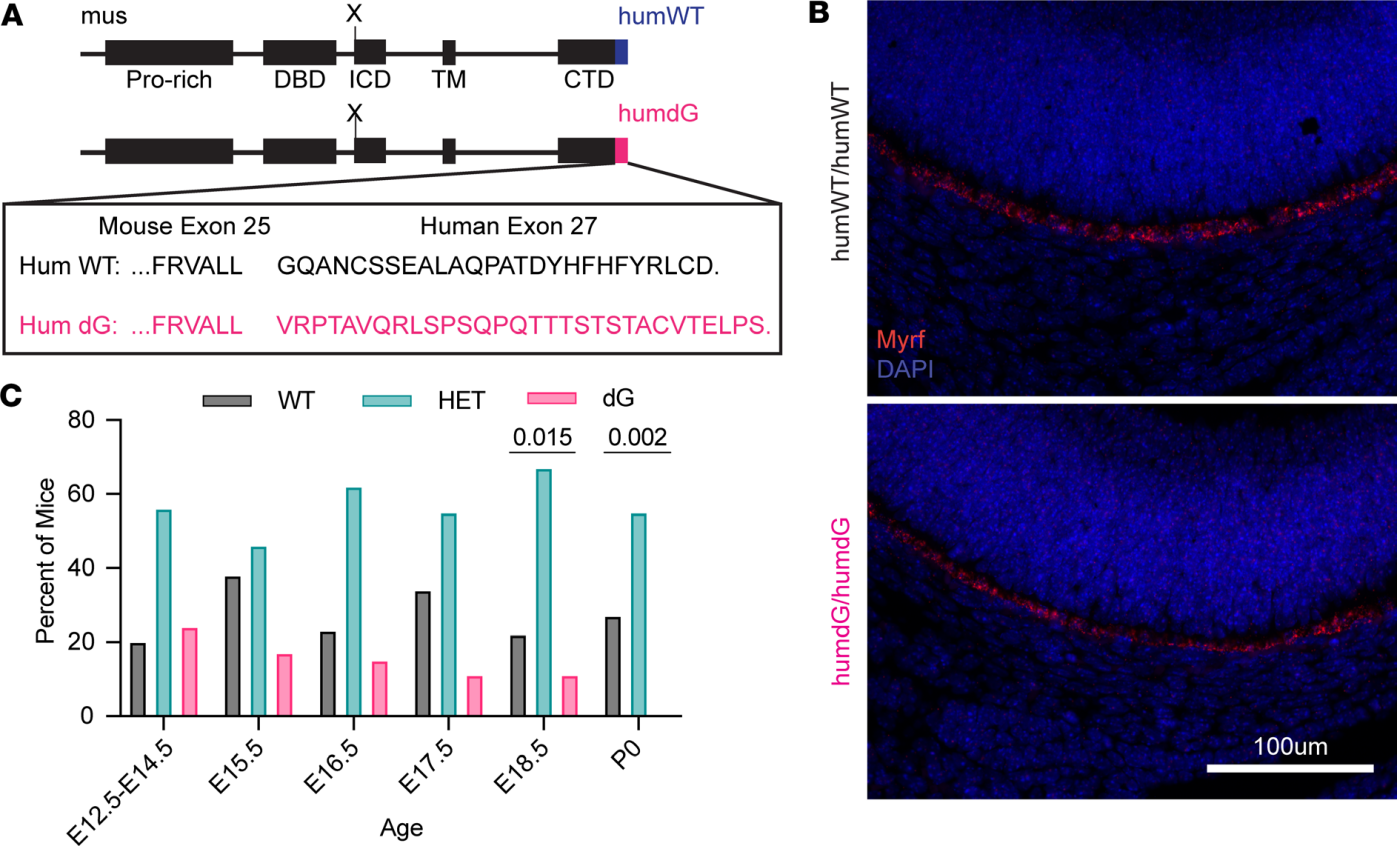
Homozygous *Myrf* humd*G* mice are embryonic lethal. (**A**) Schematic showing the (humdG) fusion allele amino acid sequence changes in the humanized mouse model. (**B**) In situ hybridization using RNAscope detected *Myr*f mRNA in humanized humWT and humdG mouse RPE (*n* = 4–5 per genotype). Scale bar: 100 μm. (**C**) Progeny from intercrossing *Myr^fhumWT/humdG^* mice exhibit skewed Mendelian ratios at E18.5 (χ^2^ *P* = 0.015) and P0 (χ^2^ *P* = 0.002). E12.5–E14.5 (*n* = 35 mice from 4 litters), E15.5 (*n* = 24 mice from 3 litters), E16.5 (*n* = 26 mice from 3 litters), E17.5 (*n* = 44 mice from 5 litters), E18.5 (*n* = 24 mice from 3 litters), and P0 (*n* = 56 mice from 5 litters).

In vitro analysis of the dG-MYRF variant (Figures 1 and 2) provided insight into the potential molecular mechanism of the patient variant *MYRF* p.Gly1126fs30*. To understand the pathogenesis of nanophthalmos in patients with the *MYRF* p.Gly1126fs30* variant, we used CRISPR/Cas9 homology–directed repair to generate a humanized mouse model that mimics the frameshift variant detected in patients, as well as a matched humanized WT control (Figure 3A). In this model, we replaced mouse exon 26 with either the human WT or human C-terminal frameshift exon 27 DNA sequence, including the endogenous-3′UTR sequence, and fused this with mouse exon 25 (Supplemental Figure 3). We previously showed that *Myrf* is highly expressed in RPE via qPCR of RNA from optic cups and RNAscope in situ hybridization (8, 10). To confirm proper cell specificity and expression of the humanized alleles of *Myrf*, we used a validated *Myrf* RNAscope probe to evaluate *Myrf* expression in *Myrf^humdG/humdG^* and *Myrf^humWT/humWT^* eyes at E15.5. These studies showed consistent *Myrf* expression pattern and levels among *Myrf^humWT/humWT^* and *Myrf^humdG/humdG^* eyes within the RPE (Figure 3B). To further define the function of the dG allele, we first evaluated survival of *Myrf^humdG/humdG^* embryos. We observed that homozygous *Myrf^humdG/humdG^* embryos were never detected after birth, while *Myrf^humWT/humWT^* mice were viable and fertile. *Myrf^humdG/humdG^* embryos were present in normal Mendelian ratios at E12.5–E17.5 but underrepresented at E18.5 (χ^2^ prob = 0.015) and at birth (P0) (χ^2^ prob = 0.042) (Figure 3C).

Analysis of ocular histology in E16.5 homozygous *Myrf^humdG/humdG^* mice revealed normal pigmentation, which differed from the severe and early-onset depigmentation phenotype we observed in our *RxCre;Myrf^fl/fl^* mice (Supplemental Figure 4) (8). *Tmem98*, a direct target of MYRF, is expressed at similar levels in control eyes (*Myrf^humWT/humWT^*) and *Myrf^humdG/humdG^* eyes at the RNA and protein level (Supplemental Figure 5) (8, 26). We cannot rule out the possibility that there are small expression differences not detected by the mRNA and immunostaining assays. However, the *RxCre;Myrf^fl/fl^* eyes had profoundly reduced expression of *Tmem98* (10). The lethality of *Myrf^humdG/humdG^* mice confirm the pathogenicity of the C-terminal *Myrf* mutant allele, and the lack of obvious ocular defects suggest that it likely functions as a hypomorphic (partial loss-of-function allele) rather than null allele.

### Myrf^humdG/+^ have normal eye size, retinal and RPE morphology, and retinal function.

**Figure 4:**
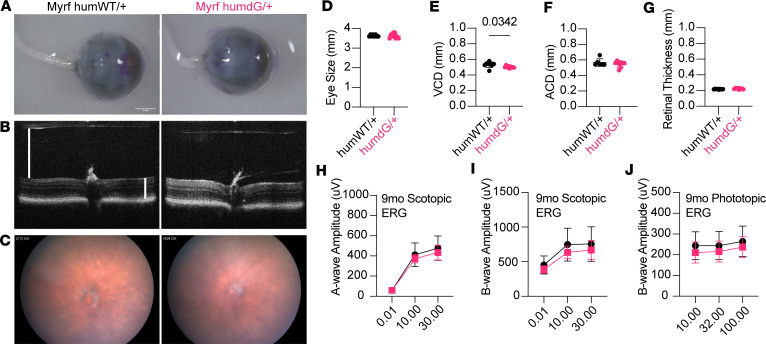
Heterozygous *Myr^fhumdG/+^* mice show no gross morphological or retinal eye phenotype after 1 year. (**A**) Images of enucleated eyes showing similar eye size. Scale bar: 1 mm. (**B**) Spectral domain optical coherence tomography (SD-OCT) images showing no change in overall thickness or individual retinal layers with white lines representing measurements for vitreous chamber depth (VCD) and retinal thickness. (**C**) Fundus photographs showing no signs of retinal white spots in *Myrf^humdG/+^* (pink) compared with controls (black). (**D**–**G**) Quantitative assessment of eye size (**D**), VCD (**E**), anterior chamber depth (**F**), and retinal thickness (**G**) by Student’s *t* test. (**H**–**J**) Retinal function measured by scotopic electroretinogram (ERG) (**H** and **I**) and photopic ERG (**J**) showed no differences (*n* = 6–8 per genotype). Scale bar: 1 mm.

The *RxCre;Myrf^fl/+^* mice had white spots in the retina and *RxCre;Myrf^fl/fl^* mice had severe retinal degeneration, though no measurable eye size phenotype was observed in either genotype into early adulthood (8). We systematically profiled *Myrf^humWT/+^* and *Myrf^humdG/+^* mice over the course of 1 year to determine whether changes ocular dimensions, retinal structure, or function would emerge over time. Noninvasive imaging (SD-OCT and fundus photography) and electroretinography (ERG) were carried out on the same mice over the course of 1-year at 3-month intervals. After 1 year, *Myrf^humdG/+^* show no significant difference in eye size, axial length, corneal diameter, anterior chamber depth, or retinal thickness and only a modest decline in vitreous chamber depth compared with controls (0.53 ± 0.03 mm versus 0.50 ± 0.01 mm, *P* = 0.0342) (Figure 4, A–G). Additionally, scotopic and photopic ERGs showed no decrease in visual function, consistent with the SD-OCT findings of normal retinal thickness and morphology (Figure 4, H–J). Fundus photography showed little to no signs of atrophy after 1 year (Figure 4, B and C). To investigate for more subtle changes to RPE morphology similar to those that were evident in *RxCre;Myrf^fl/fl^* eyes (8), RPE flatmounts were stained with phalloidin, segmented using REShAPE software (27), and evaluated for morphometric features (Supplemental Figure 6). These analyses reveal no differences in the median cell area (*P* = 0.899), aspect ratio (*P* = 0.847), or hexagonality (*P* = 0.7568) of 1-year-old *Myrf^humdG/+^* eyes compared with *Myrf^humWT/+^* controls. There was also no change in the median number of neighboring RPE cells between genotypes.

To evaluate for more subtle transcriptomic changes in *Myrf^humdG/+^* compared with WT mice, we performed qPCR in P21 adult mouse optic cups including the retina and RPE (*n* = 4–9 pairs of eyes per genotype). In agreement with the lack of phenotypic changes in these mice, we found no significant differences in gene expression between *Myrf^+/+^* and *Myrf^humdG/+^* mice in key RPE genes and previously defined MYRF target genes (10) including *Mitf*, *Ermn*, *Myrf*, *Sox10*, or *Tmem98* (Supplemental Figure 7). Together, these results suggest that, at both histologic and the molecular level, *Myrf^humdG/+^* mice are similar to their matched controls.

To determine the contribution to disease pathogenesis of the *Myrf* *^humdG^* allele, we generated *RxCre;Myrf^humWT/humWT^*, *RxCre;Myrf^flhumWT^*, and *RxCre;Myrf^fl/humdG^* mice for observational studies over a 6-month period to see the effect of the humdG allele on a sensitized background with 1 allele of *Myrf* missing (Supplemental Figure 8), but the numbers were limited due to poor breeding and survival (*n* = 4 eyes, 2 mice per genotype). Despite limited sample size, the effect was so severe that it was clear that *RxCre;Myrf^fl/humdG^* showed signs of RPE degeneration compared with *RxCre;Myrf^humWT/humWT^* and *RxCre;Myrf^fl/humWT^* controls as early as 2 months and more strikingly by 6 months (Supplemental Figure 8A). In addition, *RxCre;Myrf^fl/humdG^* mice showed significant reduction in vitreous chamber depth at 4 months (0.4 ± 0.2 mm) versus *RxCre;Myrf^fl/humWT^* (0.54 ± 0.01 mm, *P* = 0.0163) and *RxCre;Myrf^humWT/humWT^* (0.525 ± 0.008 mm, *P* = 0.0106), and 6 months (0.38 ± 0.03 mm) versus *RxCre;Myrf^fl/humWT^* (0.486 ± 0.005 mm, *P* = 0.0130) and *RxCre;Myrf^humWT/humWT^* (0.54 ± 0.01 mm, *P* = 0.0013) (Supplemental Figure 8, B and D). No differences were observed between genotypes in total retinal thickness or overall eye size (Supplemental Figure 8, E and F). In addition to anatomical changes, we observed molecular changes such as mislocalization of a partner protein TMEM98 in 6-month-old RPE flat mounts from *RxCre;Myrf*^fl/humdG^ mice, which was not seen in controls (Supplemental Figure 8C). These data support the *Myrf ^humdG^* allele as a partial loss-of-function allele.

### Single-cell RNA-seq shows altered RPE gene regulatory networks in Myrf^humdG/humdG^ eyes.

**Figure 5:**
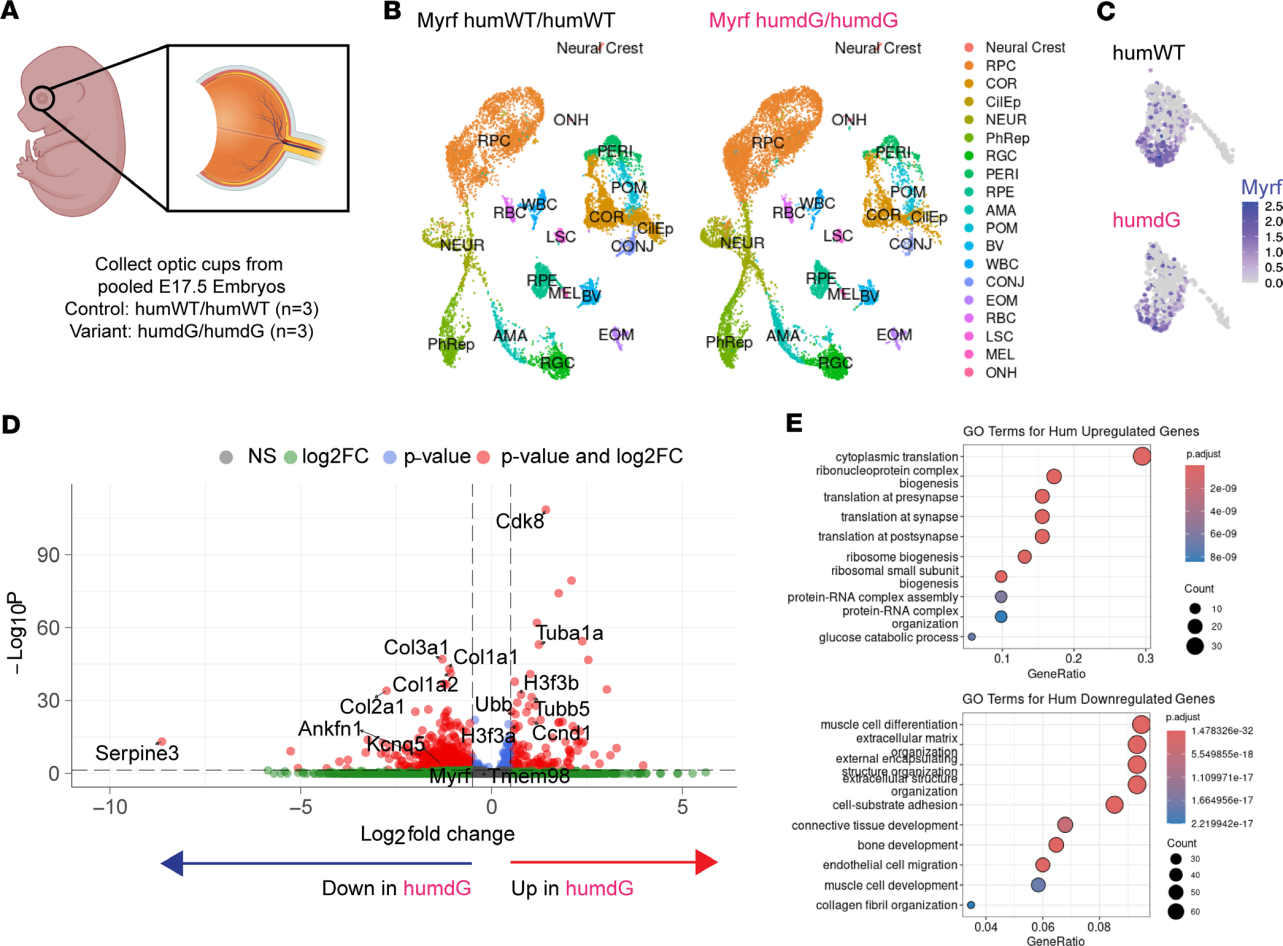
scRNA-seq reveals molecular changes in RPE of mice carrying C-terminal *Myrf* Allele (M*yrf^humdG/humdG^*). (**A**) Schematic depicting optic cups and samples used for scRNA-seq including control and variant mouse optic cups (*n* = 3 pooled per genotype). (**B**) UMAPs of control versus *Myrf* C-terminal variant mouse optic cups demonstrating cell type distributions. (**C**) Feature plot demonstrating expression of *Myrf* mRNA in both control and variant mice within the RPE cluster. (**D**) Volcano plot of differentially expressed genes in *Myrf^humdG/humdG^* mice compared with control (Myrf^humWT/humWT^). DEGs were defined by a Bonferroni *P*_adj_ < 0.1. (**E**) Top 10 Gene Ontology (GO) Biological Pathway terms for upregulated and downregulated genes.

To better understand how the C-terminal MYRF variant affects eye development, we performed single-cell RNA-seq (scRNA-seq) on E17.5 optic cups of *Myrf^humWT/humWT^* and *Myrf^humdG/humdG^* mice using the 10X genomics platform (Figure 5A). We chose E17.5, as it is occurs after our RPE pigmentation phenotype (E15.5) is observed in RxCre;Myrf^fl/fl^ mice (10) and just before Mendelian ratios become skewed at E18.5. We collected 11,281 and 11,322 cells for *Myrf^humWT/humWT^* and *Myrf^humdG/humdG^*, respectively. The median genes per cell were comparable in WT (2,429) and variant (2,415) samples. Quality control filtering was performed to filter out dead cells, doublets, and poor-quality cells (10). After integration with previously published datasets from conditional KO Myrf eyes (*RxCre;Myrf^fl/fl^* and *Myrf^fl/fl^*) at E15.5 and P0, we performed unsupervised clustering and were able to identify 19 clusters, including all the major cell types within the optic cup using previously established cell type–specific markers (Supplemental Figure 9) (10). All clusters were present in both the *Myrf^humWT/humWT^* and *Myrf^humdG/humdG^* mice (Figure 5B), but there was a slight reduction in the RPE cell proportions in *Myrf^humdG/humdG^* mice (5.0%) compared with control (6.4%) (Supplemental Figure 9, C and D).

*Myrf* expression was detected exclusively in the RPE cluster (Figure 5C), consistent with previous results, leading us to focus our downstream analysis on the RPE cluster. We performed differential gene expression (DEG) analysis between *Myrf^humWT/humWT^* and *Myrf^humdG/humdG^* RPE clusters. We found 735 upregulated and 170 downregulated differentially DEGs with avg_log_2_FC > ± 0.25 and *P*_adj_ ≤ 0.1 (Figure 5D), *Myrf^humdG/humdG^* relative to *Myrf^humWT/humWT^* controls. Downregulated genes included extracellular matrix–related (ECM-related) genes C*ol3a1*, *Col2a1*, *Col1a1*, and *Col1a2* produced by the RPE that play an important role in structure and barrier maintenance (28). Interestingly, some of the most downregulated genes in the variant mice are related to eye size disorders including *Serpine3* (avg_log_2_FC = –1.11, *P*_adj_ = 2.76E-14), *Ankfn1* (avg_log_2_FC = –1.138, *P*_adj_ = 2.72 × 10^–9^), and *Kcnq5* (avg_log_2_FC = –0.81, *P*_adj_=6.39 × 10^–5^) (29–33). Upregulated genes included cell cycle genes *Ccnd1* and *Cdk8*, histone variant components *H3f3b* (avg_log_2_FC = 0.71, *P*_adj_ = 3.69 × 10^–34^) and *H3f3a* (avg_log_2_FC = 0.56, *P*_adj_ = 3.30 × 10^–23^), and ubiquitin protein ligase binding genes *Tubb5*, *Ubb* (avg_log_2_FC = 0.59, *P*_adj_ = 2.32 × 10^–27^), *Tuba1a* (avg_log_2_FC = 0.32, *P*_adj_ = 0.03). We used overrepresentation analysis (ORA) to identify the top 10 gene ontology terms for Biological Processes and molecular function pathways in humanized variant mice (*Myrf^humdG/humdG^*) and conditional KO (*RxCre;Myrf^fl/fl^*) relative to their matched controls (*Myrf^humWT/humWT^* and *Myrf^fl/fl^*). We looked for overlap in top GO terms enriched in *Myrf^humdG/humdG^* and condition KO compared with controls and found shared downregulation of ECM organization (GO:0030198) and extracellular structural organization (GO:0043062). In addition, shared upregulated pathways included structural constituent of ribosome (GO:0003735), structural molecule activity (GO:0005198), rRNA binding (GO:0019843), and translation (GO:0006412) (Figure 5E). Furthermore, GO analysis identified many upregulated pathways in both humanized and KO mice related to metabolism, mitochondrial, and ribosomal function. Thus, the humanized C-terminal variant mutant (*Myrf^humdG/humdG^*) resulted in RPE transcriptome changes similar to the conditional KO (*RxCre;Myrf^fl/fl^*), including key pathways involved in RPE maintenance and development.

**Figure 6:**
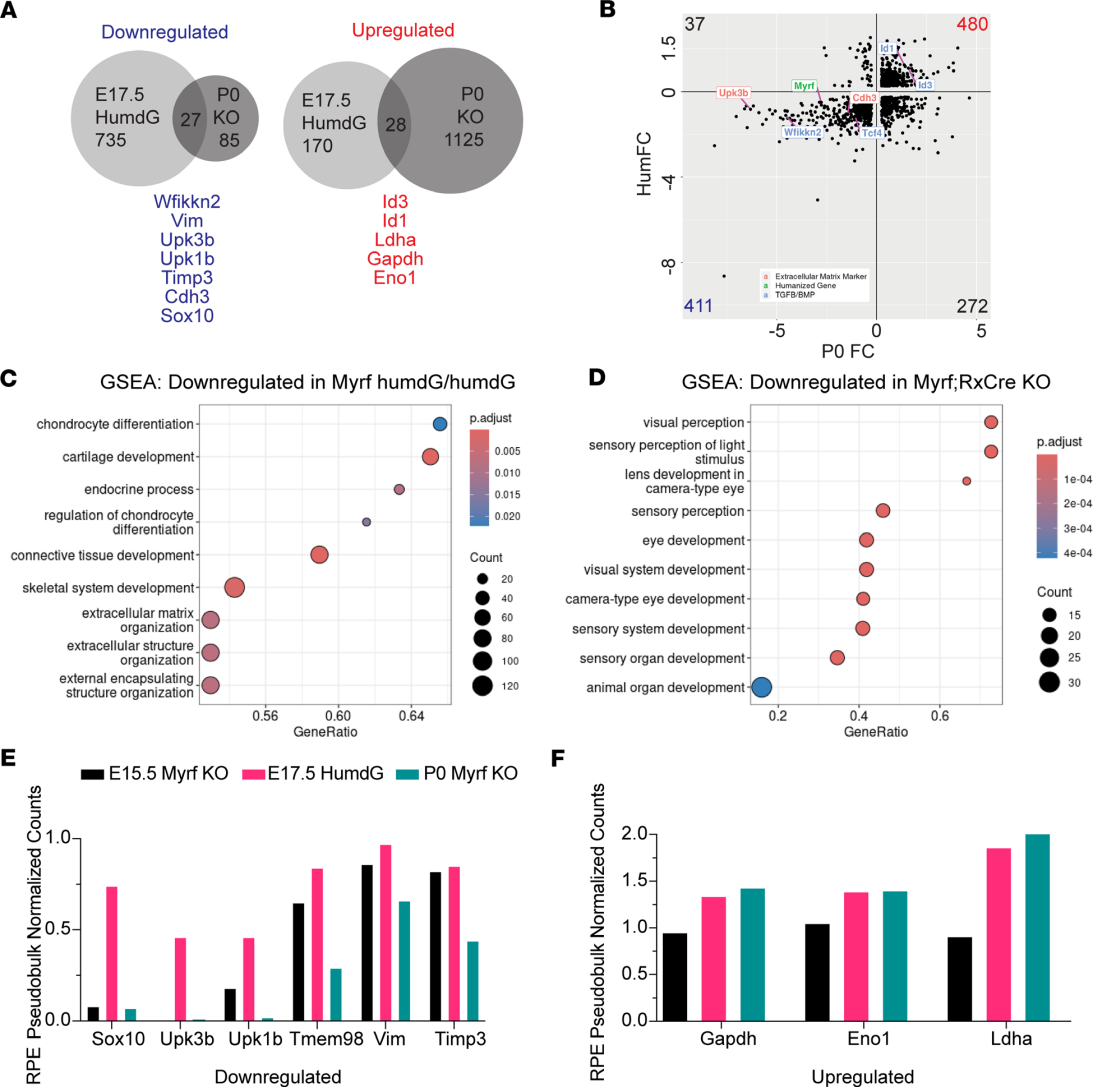
*Myrf^humdG^* C-terminal variant acts as a hypomorphic allele. (**A**) Comparison of differentially expressed genes in RPE cluster of E17.5 humanized C-terminal variant (*Myrf^humdG/humdG^*) versus conditional KO (*RxCre;Myrf^fl/fl^*) mice with key shared genes highlighted (*n* = 3 pooled per genotype). (**B**) Plot of *Myrf^humdG/humdG^* versus *RxCre;Myrf^fl/fl^* fold change in gene expression for genes expressed in both datasets (log_2_FC > 0.25, Bonferroni *P*_adj_ < 0.1) showing strong concordance of up- and downregulated differentially expressed genes between the humanized allele and conditional KO. (**C** and **D**) Gene set enrichment analysis (GSEA) showing top 10 downregulated pathways in *Myrf^humdG/humdG^* versus *RxCre;Myrf^fl/fl^* mice. (**E** and **F**) Pseudobulk analysis of RPE cluster demonstrating magnitude of downregulation compared with respective control (**E**) is lower in the C-terminal variant (pink) than the conditional knock out at E15.5 (black) or P0 time points (blue), while magnitude of upregulation is similar across groups (**F**).

We extended the DEG analysis of the humanized C-terminal variant allele (*Myrf^humdG/humdG^*) and conditional KO (*RxCre;Myrf^fl/fl^*) by examining individual genes that were affected, including the direction and magnitude of the effect. After integration, we performed DEG analysis on these 2 conditions and looked for genes that had an avg_log_2_FC ≥ abs(0.25) and *P*_adj_ ≤ 0.1 in both the C-terminal variant and conditional KO mice compared with controls. We found a total of 55 shared DEGs, with 27 downregulated and 28 upregulated genes respectively (Figure 6A and Supplemental Table 1). Key genes important for RPE development and maintenance were concordant, but the magnitude of the change was decreased in *Myrf^humdG/humdG^* as compared with *RxCre;Myrf^fl/fl^* mice (Supplemental Figure 10). Some of the concordant downregulated genes and pathways included ECM matrix (*Upk3b*, *Upk1b*, *Cdh3*), Wnt signaling (*Tcf4*), TGF-β inhibition (*Wfkkin2*), and *Myrf*. The concordance of upregulated genes included increases in TGF-β signaling (*Id1* and *Id3*) and glucose metabolism (*Gapdh*, *Ldha*, *Eno1*) (Figure 6B). To validate our findings from that scRNA-seq dataset, we performed qPCR on E16.5 whole eyes (Supplemental Figure 11). We were able to confirm statistically significant increases in expression of *Gapdh* (1.16 *±* 0.26 versus 0.93 *±* 0.31 fold change, *P* = 0.0481) and *Ldha* (1.33 *±* 0.19 versus 0.939 *±* 0.19 fold change, *P* = 0.0267) in Myrf^humdG/humdG^ mice compared with controls (*n* = 7–8 pairs of eyes per genotype). Gene set enrichment analysis (GSEA) was performed separately on both *Myrf^humdG/humdG^* and *RxCre;Myrf^fl/fl^* paired with matched controls, *Myrf^humWT/humWT^* and *Myrf^fl/fl^*, respectively. GSEA results from *Myrf^humdG/humdG^* RPE emphasized downregulation of ECM and bone development/differentiation pathways, consistent with our prior reports, which showed similar gene enrichment in *RxCre;Myrf^fl/fl^* RPE. Notably, pathways related to eye and sensory organ development were absent in the top GSEA results for *Myrf^humdG/humdG^*, while they dominated the top results for *RxCre;Myrf^fl/fl^* (Figure 6, C and D).

Finally, to assess the retained function of the humdG allele relative to conditional KO, we performed pseudobulk analysis on the RPE clusters and compared expression of select downregulated (Figure 6E) or upregulated (Figure 6F) genes between *Myrf^humdG/humdG^* (E17.5) and *RxCre;Myrf^fl/fl^* (E15.5 and P0). We observed that the *Myrf^humdG/humdG^* RPE retained 46%–90% expression across various downregulated genes as compared with P0 *RxCre;Myrf^fl/fl^* mice, which only retains 1%–66% expression as compared with their respective controls. Interestingly, for select upregulated genes, we observed no differences in expression between the genotypes, suggesting these are less likely to be direct targets and may be buffered. Together, these data support shared pathogenic mechanisms between the KO model and the C-terminal variant, stemming from hypomorphic MYRF effects at the level of RPE.

### Deep intronic variants in MYRF in nanophthalmos patients alter splicing.

**Figure 7:**
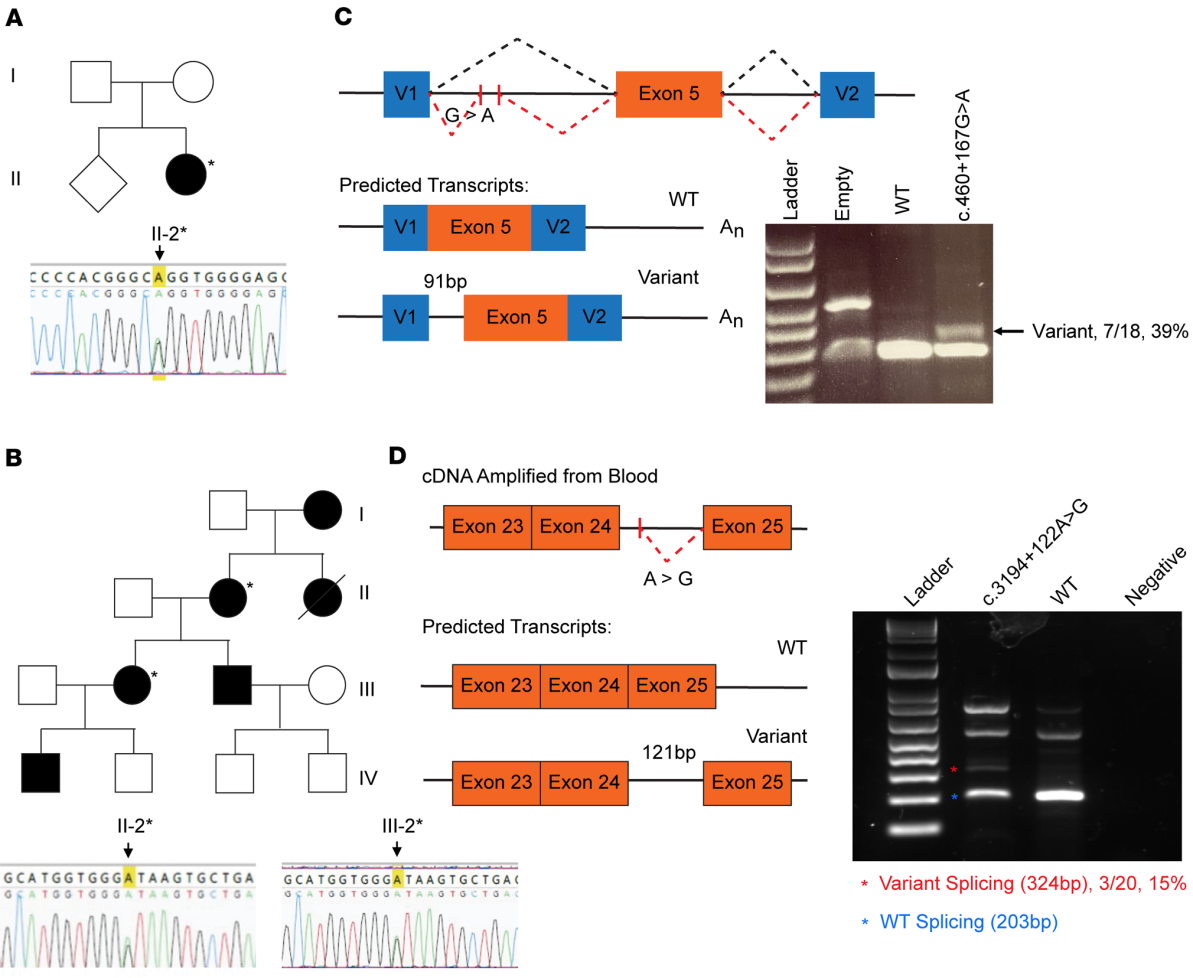
Deep intronic variants in *MYRF* alter splicing of mRNA transcript. (**A** and **B**) Two families were discovered with intronic variants in *MYRF*. (**C**) Schematic of the minigene RNA splicing assay and predicted products. Minigene for variant c.460+167G>A (**A**) show altered splicing of *MYRF* at the mRNA level for 39% of transcripts from sample from Family 1, suggesting a ~19% usage overall (*n* = 18 colonies). (**D**) cDNA and predicted splicing of c.3194+122A>G (**B**) variant amplified from *MYRF* RNA in patient blood from Family 2. PCR amplification of cDNA shows altered splicing at the mRNA level for 15% of transcripts (30% for the allele) (*n* = 20 colonies).

As our in vitro and transcriptomic findings suggest a hypomorphic effect for the dG-MYRF variant, we reasoned that other *MYRF* splicing variants may also cause ocular only phenotypes, either by affecting only the C-terminal domain exons or by causing a partial reduction in functional RNA or protein products. To validate this hypothesis, panel-based or whole genome sequencing (WGS) was carried out on a cohort of 53 families with nanophthalmos/high hyperopia that did not have identified genetic diagnoses (34). From this cohort, 2 families were identified with deep intronic variants in *MYRF* (NM_001127392.3 MYRF c.460+167G>A in P01965 from Family 1 and c.3194+122A>G in P04818 and P04825 from Family 2) (Figure 7, A and B). No other rare deleterious genetic variants in nanophthalmos-associated genes (*MYRF*, *PRSS56*, *MFRP*, *CRB1*, BEST1) were identified in either family.

Clinical examination of family members revealed very short axial lengths in both families and classic features of nanophthalmos including choroidal folds and retinal detachment (Supplemental Figure 9 and Supplemental Table 2). Proband P01965 demonstrated no family history of nanophthalmos (Figure 7A), while Family 2 exhibited a pattern of autosomal dominant inheritance, with demonstrated segregation in 2 generations. No systemic features of MYRF cardiac-urogenital syndrome were noted in any of the affected individuals or other family members.

The high spliceAI (35) scores for the variants in Family 1 and 2 (c.460+167G>A, score = 0.992 and c.3194+122A>G, score = 0.8, respectively) indicate that the variants have a high probability of altering splicing (Supplemental Table 3). The first variant was predicted to cause gain of a cryptic acceptor site (c.460+167G>A), leading to pseudoexon insertion of 91 bp (Figure 7C) and a frameshift causing either early protein truncation or RNA degradation via nonsense mediated decay. An exon trap minigene splicing assay confirmed that the c.460+167G>A variant altered splicing compared with WT *MYRF*, producing a 374 bp fragment corresponding to the predicted pseudoexon inclusion in addition to the normal splicing product (283 bp WT exon 5 amplicon) (Figure 7C). To quantify the fraction of transcripts that have aberrant splicing, PCR fragments were TA cloned, and individual colonies were sequenced (*n* = 18). Seven of 18 (38.9%) contained RNA produced from the cryptic splice site usage, while the remaining used the normal splice acceptor.

For Family 2, we confirmed cosegregation of the variant in 2 generations, yet we did not detect pseudoexon inclusion using the exon trap vector system (Supplemental Figure 13, A–C). The variant may have a cell type–specific or contextual effect on splicing not detected by our assay or affect *MYRF* function through a different mechanism. To support this possibility, we extracted RNA from freshly collected patient blood and performed RT-PCR to look for evidence of altered transcript splicing. We observed an additional ~300 bp band matching the expected size of an alternatively spliced transcript for this variant (Figure 7D). To quantify the fraction of transcripts that have aberrant splicing, PCR fragments were TA cloned, and individual colonies were sequenced, showing 3 of 20 colonies (15%) using the cryptic splice site usage, and suggesting ~30% usage of the cryptic splice site from that allele overall (Figure 7D and Supplemental Figure 13D). The predicted protein product based on cDNA sequencing would cause a frameshift leading to early truncation after exon 24 (Supplemental Figure 13E).

Together, these data suggest that intronic splice variants in *MYRF* may be another mechanism leading to the pathogenesis of nanophthalmos. Isolated ocular phenotypes may be driven by partial loss-of-function alleles with incomplete alternative splice site compensation or altered function of the alternative transcript’s protein product.

## Discussion

**Figure 8:**
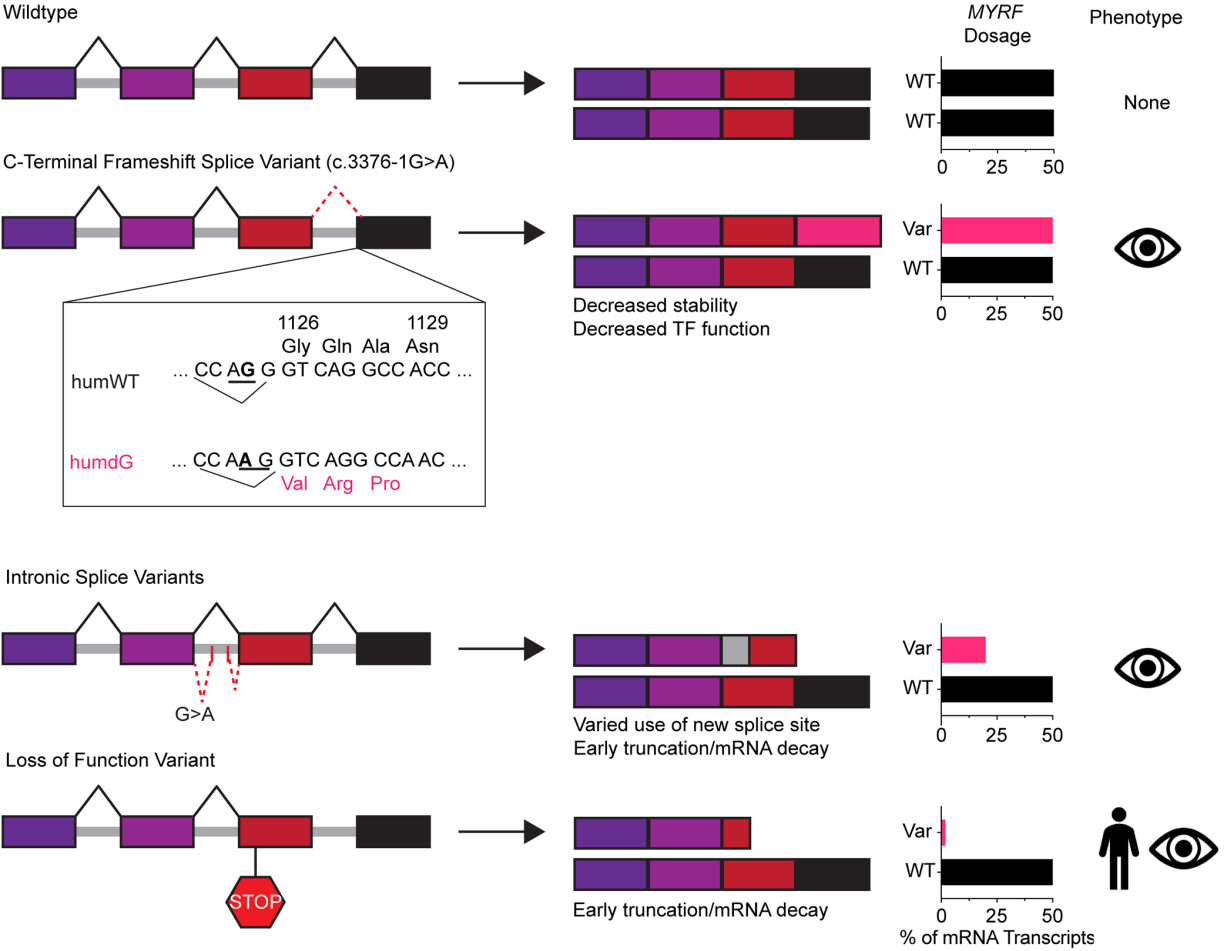
Working model of *MYRF* splice variant functional and phenotypic effects contributing to isolated ocular and syndromic disease pathogenesis. WT *MYRF* retains transcription factor function resulting in no phenotypic effects. A C-terminal frameshift splice variant results in a 31 amino acid frameshift leading to decreased C-terminus stability and transcriptional activation, leading to reduced transcription factor function and isolated ocular disease. Intronic splicing variants lead to a frameshift that results in early truncation or nonsense mediated RNA decay. These splice sites are not always used, leading to modest decrease in MYRF transcription factor function and isolated ocular disease. In contrast, complete loss of function alleles either lead to early truncation/nonsense-mediated decay or alter critical functional domains (i.e., DNA binding, cleavage) and lead to severe, syndromic symptoms.

Though originally identified as a master regulator of oligodendrocyte maturation, growing evidence supports a role for MYRF in regulating RPE development, differentiation, and maturation (8, 10). Our previous studies underscore the importance of MYRF in the developing RPE, showing that conditional loss of *Myrf* in mice can be disastrous for RPE development and lead to retinal degeneration (8). Interestingly, there are a cluster of variants located in the conserved C2 domain that manifest as isolated ocular diseases (13). Combined with other partial loss-of-function alleles, including splicing variants we further identify in this study, our data support that the C2 domain variants and other hypomorphic alleles may manifest as isolated ocular disease (Figure 8).

### C-terminal MYRF variant acts as a loss-of-function allele.

We show 4 lines of evidence supporting that the C-terminal MYRF variant is a partial loss-of-function allele. First, embryonic lethality is observed in mice harboring a homozygous C-terminal truncating allele in *Myrf*, in line with the autosomal dominant inheritance pattern and lack of homozygous mutant individuals in humans. Embryonic lethality is observed later in gestation than constitutional KO of *Myrf*, with skewed Mendelian ratios starting at E17.5, rather than lethality during cardiac development midgestation (36). Second, there are many shared disrupted transcriptional targets and pathways, in our *Myrf^humdG/humdG^* mouse model and the *RxCre;Myrf^fl/fl^* conditional KO mice, including ECM, TGF-β signaling, Wnt signaling, structural regulation, and RPE maturation pathways, and regulation of *Myrf* itself, arguing against neomorphic function.

Third, transcriptional and phenotypic effects in *Myrf^humdG/humdG^* were milder than in comparable *RxCre;Myrf^fl/fl^* mice. *Myrf^humdG/humdG^* had normal-appearing RPE pigmentation, modest reduction in *Tmem98* expression, and lower magnitude of DEGs among shared pathways with the *RxCre;Myrf^fl/fl^* eyes. Additionally, no phenotypic changes in the heterozygous *Myrf^humdG/+^* eyes were apparent over 1 year. The direct comparison of scRNA-seq datasets with the conditional KO suggests that the C-terminal *MYRF* variant allele retains some partial function. Although molecular changes are picked up on scRNA-seq data, the phenotypic changes in the *Myrf^humdG/humdG^* are more modest in comparison with the *RxCre;Myrf^fl/fl^* mice. For example, genes in the pigmentation pathway are present in the downregulated DEGs of *Myrf^humdG/humdG^* such as *Oca2* at E17.5, but these mice show no signs of depigmentation at embryonic stages. Fourth, the C-terminal frameshift *MYRF* allele exhibits only subtle processing defects, with decreased steady state stability and transcriptional activity in vitro, but no differences in protein, localization, or cleavage in the context of an overexpression assay.

A previous study developed a mouse model to investigate a similar variant, *MYRF* c.3260delG/p.(Gly1087Valfs*151) mutation, which corresponds to the human *MYRF* c.3377delG/p.(Gly1126Valsf*31) mutation (9). This mutation causes the same C-terminal frameshift in humans as the c.3776-1G>A discovered by our lab. While there was support for loss-of-function for this allele, with lower mRNA and protein expression of MYRF (9), the mouse allele did not mimic the human allele, given the difference in the frameshift amino acid sequence and the-3′UTR sequence. Furthermore, comparison of embryonic lethality of the Gly1087Valfs*151 variant allele to our current study is not possible, as the prior study only evaluated heterozygous mice. Therefore, our humanized mouse has more fidelity to model the human disease by replacing the last exon with human WT or variant DNA sequence and recapitulates the 31 amino acid frameshift in patients. Some discrepancies remain between our model and the previously published one (9), as we did not find evidence to support changes in thickness of GCL or INL layers (measured by SD-OCT), phenotypic/morphological changes in heterozygous mice, or signs of primary angle closure glaucoma. These may be related to the different nature of the allele, CRISPR off-target effects, or strain/background specific effects. Our humanized mice and WT mice exhibit mRNA expression exclusively within the RPE cluster; therefore, antibody detection of MYRF in other eye structures may represent cross-reactivity or background (8, 10). Our data, together with these previous models, support the assertion that C-terminal *Myrf* variant is acting as a partial loss-of-function allele. However, future studies are necessary to completely rule out the possibility of the C-terminal variant acting via dominant negative interactions with the WT MYRF, as these mechanisms could not be assessed easily in our animal model or overexpression cell culture models.

### Eye size and the dG-MYRF mouse model.

In both our animal model of the human allele and our prior developmental KO model (8, 10), axial lengths were comparable with those of the control, suggesting either that the mouse may not recapitulate all the features of human nanophthalmos or that more efficient KO or longer follow-up is necessary to see this phenotype. The 2 mouse models of recessive nanophthalmos genes, *MFRP* and *PRSS56*, with the most severe axial length reductions in humans (34, 37, 38) have produced only modest reductions in axial length (<0.15–0.2 mm) (39–41). Mouse models in other nanophthalmos-associated genes, including *BEST1*, *CRB1*, and *TMEM98* do not show reductions in axial length (42–46) but do show various degrees of reduced retinal function and/or retinal degeneration, consistent with our models of *Myrf* (40). Furthermore, all 3 of the RPE-derived genes (*Best1*, *Tmem98*, and *Myrf*) are expressed in adult RPE, suggesting a potential maintenance role in RPE. Complex molecular signaling between the RPE and retina may lead to compensation in ocular dimensions, leading to no observable changes in eye size. For example, upregulation of *Prss56* is observed in *Mfrp* rd6-KO mice and AAV2 mediated overexpression of *Prss56* alone in adult mouse eyes can increase axial length (41). Thus, *Prss56* upregulation may temper any reduction in eye size in the *Mfrp-*KO mouse. Interestingly, in our cohort of *RxCre;Myrf^fl/humdG^* mice we saw limited effects on retinal thickness but observed a significant decrease in VCD after 4 and 6 months compared with either *RxCre;Myrf^fl/humWT^* or *RxCre;Myrf^humWT/humWT^*. Changes in VCD are correlated to eye size and refractive error in humans and mice (47–50). Measurements of overall eye size may be complicated by the technique and variability, and future studies will be necessary to fully evaluate the contribution of MYRF to eye size phenotypes in mice. Finally, our data suggest that eye size defects may precede retinal degeneration, which was seen more robustly in our *RxCre;Myrf^fl/fl^*–conditional KO model. Further work to explore the compensatory mechanisms buffering eye growth will be informative to address the discrepancies between human disease and mouse models.

### Consequences of functional loss of C-terminal domain.

Our results show that the C-terminal MYRF variant can cleave and localize properly to the nucleus like the WT protein. This is consistent with previous work that demonstrated a truncated version of MYRF lacking the C-terminal domain (MYRF-1:756) maintains proper homotrimerization and processing in oligodendrocytes (2). Previous work has also shown that homotrimerization is necessary for MYRF self-cleavage (1). This implies that the C-terminal variant is also able to form the homotrimer structure necessary to facilitate cleavage of MYRF. As the C-terminal MYRF variant homotrimerizes and cleaves, we might expect it to be capable of activating transcription of its downstream targets. However, our scRNA-seq and qPCR data show downregulation of key target genes and pathways involved in RPE development including the Wnt signaling, TGF-β signaling, pigmentation, cytoskeletal organization, and epithelial cell development. The changes in these pathways are consistent with many changes detected in our conditional KO model. For example, top downregulated hits in MYRF C-terminal variant RPE included known eye size disorder genes. *Serpine3* encodes a serine protease inhibitor, which has been shown to be significantly upregulated in induced myopia models in zebrafish and chicks (30, 31). In contrast, it is significantly reduced in our model, suggesting that it may play a bidirectional role in eye size regulation like *Prss56*. Additionally, other top downregulated genes *Ankfn1* and *Kcnq5* are known GWAS hits for refractive error and/or myopia (51–53). Downregulated *Ankfn1* mRNA expression has recently been discovered as a defining feature of healthy versus dedifferentiated RPE in mouse models that are aged or exposed to cigarette smoke (29). *Kcnq5* encodes a voltage-gated potassium channel expressed in the RPE (human, primate, and bovine) and involved in active transport of K^+^ from the retina to the choroid (32, 33). These data suggest that the C-terminal variant is likely influencing expression of genes involved in eye size regulation in the RPE and demonstrates how leveraging scRNA-seq technology can help to uncover early changes in gene expression, which are challenging to detect via bulk RNA-seq or qPCR in complex tissues like the developing embryonic eye.

Interestingly, although dG-MYRF maintains normal protein processing, at steady state, the full-length and C-terminal fragments are less stable. It is possible that the instability observed at steady state levels may be a driving factor behind decreased transcriptional activation of downstream target genes. Furthermore, with in vitr*o* overexpression, we see loss of *MYRF* autoregulation and inability to upregulate the *TMEM98* downstream target in the dG-MYRF mutant. These results are consistent with other studies supporting a role for the C-terminus of MYRF in regulation of its function, as removal of the ER-resident portion in vitro results in decreased levels of target gene transcription (2, 54). Furthermore, overexpression of a mouse *Myrf* plasmid missing the ER portion results in decreased expression of genetic markers of differentiation in maturing oligodendrocytes (2). Together, these data suggest that the C-terminal MYRF variant and removal of the ER portion may decrease target gene expression, even though the protein appears to maintain normal cleavage and localization dynamics in cell culture, suggesting that other important protein-protein interactions may be at play.

### Presence of splice-altering variants in nanophthalmos.

In addition to variants in well-characterized domains such as the DBD and the ICA cleavage domain, which fundamentally alter MYRF’s function to act as a transcription factor, we found predicted splice variants in *MYRF* that are associated with nanophthalmos. We discovered what we believe to be two novel, deep intronic variants in *MYRF* that are predicted to alter splicing; one of these was confirmed by a minigene splicing assay and the other by directly assessing patient blood RNA for alternatively spliced transcripts. The splicing variant from Family 1 is expected to result in an early frameshift and produce a nonfunctional protein product. The minigene splicing assay suggests that pseudoexon inclusion occurs in ~40% of the transcripts from that allele. The splicing variant from Family 2 is expected to create a stop codon after exon 24 and predicted to create a truncated protein product with reduced function. Splicing from patient blood suggests that the pseudoexon inclusion occurs in ~30% of the transcripts from that allele. These data, together with our animal and cell culture evidence from the C-terminal frameshift variant, support a tissue-specific, dosage-sensitivity threshold for *MYRF*, whereby partial loss-of-function variants may lead to ocular disease only, while complete loss-of-function or dominant negative alleles may lead to syndromic disease. Dosage sensitivity of MYRF is supported by previous studies showing intrafamilial phenotypic variability (8, 17, 55). Indeed, most individuals with cardiac-urogenital syndrome that are evaluated for ocular phenotypes do have high hyperopia (3). Since WGS is applied more often in clinical settings, deep intronic variants in *MYRF* and other nanophthalmos-associated genes may be found. Furthermore, WGS may improve our ability to capture DNA variants in highly conserved noncoding regions such as promoters or enhancers that may be regulated by MYRF. One limitation is that *MYRF* variants are a rare cause of nanophthalmos and, therefore, we may not observe the exact same variants in multiple families. However, we expect WGS to reveal similar splicing alternations that lead to production of nonfunctional transcripts. Additionally, future studies could benefit from implementation of long-read sequencing to resolve additional splice isoforms (at the RNA level) or any changes in RNA modifications in *MYRF* (56) and allow for haplotype resolution and assessment of epigenetic marks (56, 57) at the DNA level.

### Conclusions.

In this study, we have investigated mechanisms by which MYRF alleles may cause isolated ocular disease. Our results emphasize the role of the protein’s C2 domain in regulating of the activity of MYRF in the RPE, as its alteration leads to decreased steady-state stability, transcriptome-wide dysregulation, and downregulation of pathways important for RPE development. We also demonstrate that the C-terminus of MYRF is important and necessary to produce viable offspring in mice. These results build upon the previously existing limited knowledge of the function and importance of the conserved C-terminal domain of MYRF and offer steppingstones to further understand how C-terminal variants of MYRF contribute to disease pathogenesis. Furthermore, our identification of variant alleles that alter splicing, while maintaining some appropriate splicing, supports the conclusion that the eye is particularly sensitive to reduced MYRF function.

## Methods

### Sex as a biological variable.

Our study examined male and female animals, and similar findings are reported for both sexes.

### Plasmid generation and lentiviral transduction of ARPE-19 cells.

Human FLAG-tagged WT-MYRF DNA constructs were generated (Twist Bioscience, South San Francisco, CA) in a pLentiLox-IRES-GFP backbone vector (58). The C-terminal MYRF mutant and the V679A cleavage deficient were generated by site-directed mutagenesis of the pLentiLox-FLAG-MYRF-IRES-GFP plasmid using partially overlapping primers (Forward: 5′ GCACTGCTGGTCAGGCCAACTGCAGTTCAGAGG-3′, Reverse: 5′-TGGCCTGACCAGCAGTGCCACCCGAAAGTGGTA-3′) and the Quik Change II Kit (Agilent Technologies, Santa Clara, CA, #200517) (Supplemental Table 5) and verified by junctional Sanger sequencing, and whole plasmid sequencing (Plasmidsaurus, South San Francisco, CA). Lentiviral constructs were packaged by the University of Michigan Vector Core (10X concentrated virus) using standard methods. Viral titers were assessed by flow cytometry for GFP on subconfluent ARPE-19 cells with assistance from the University of Michigan Flow Cytometry Core.

### ARPE-19 cell culture.

ARPE-19 cells (CRL-2302,American Type Culture Collection Manassas, VA) were grown according to established protocols (59, 60) on DMEM/F-12 HEPES media (Thermo Fisher Scientific, Waltham, MA, #11330032) supplemented with 10% fetal bovine serum (FBS) (Corning, Glendale, AZ, #35-010-CV), 10 mM nicotinamide (Sigma Aldrich, Burlington, MA, #N0636), and 1% penicillin/streptomycin (Gibco, Waltham, MA, #15140-122), and passaged 1:4 on P100 plates.

### Human primary RPE cultures and TEER.

Primary human RPE cultures (hRPE) were grown according to established protocols (24, 61–64). Cells for localization studies were seeded on passage 1 and grown on microporous inserts (Transwells [Corning, Glendale, AZ, USA], product 3470) for at least 2 months. All primary cells were genotyped for Age Related Macular Degeneration risk SNPs: CFH (Y402H, rs1061170), C3 (R102G, rs2230199), C2 (rs9332739), and HTRA1/ARMS2 (rs3750846) using PCR amplification with primers in Supplemental Table 4 followed by ExoSAP-IT PCR Product Cleanup (Thermo Fisher Scientific, Waltham, MA, #78200.200.UL) and Sanger sequencing (Eurofin, Louisville, KY). No high-risk SNPs were detected.

### Protein localization studies.

ARPE-19 cells were seeded at 40,000 cells/well on Nunc Lab-Tek II 4-well chamber slides (Thermo Fisher Scientific, Waltham, MA). After 24 hours, cells were transduced with 1 × 10^–2^ dilution of WT-MYRF, C-terminal mutant MYRF, or V679A (10X concentrated) vectors in the presence of 100 mg/mL LentiBoost (Mayflower Biosciences, St. Louis, MO, #SB-P-LV-101-11). Cells were fixed with 4% PFA (paraformaldehyde) in 1× PBS (phosphate buffered saline) for 10 min, incubated with rabbit anti-FLAG 1:1,000 (Cell Signaling Technology, Danvers, MA, #14793) and mouse anti-Calnexin 1:200 (Sigma Aldrich, Burlington, MA, #C4731) primary antibodies overnight at 4°C followed by incubation with anti-goat rabbit Alexa Fluor 488 1:500 (Jackson ImmunoResearch Labs, WestGrove, PA, #111-545-144) and goat anti-mouse Alexa Fluor 555 1:1,000 (Invitrogen, Carlsbad, CA, A-21422) secondary antibodies for 2 hours at room temperature (RT). Cells were washed 3× 5 min with 1×PBS,stained with DAPI (Thermo Fisher Scientific, Waltham,MA), mounted with gold anti-fade media (Thermo Fisher Scientific, Waltham, MA, P36930), and imaged at 63X using Leica SP8 confocal microscope (Leica, Wetzlar, Germany).

Confluent and mature hRPE were transduced at an MOI of 5 with either an Empty, WT-MYRF, or C-terminal mutant MYRF (10X concentrated) vectors in the presence of 100 mg/mL LentiBoost. After 1 week, cells were fixed with a 4% PFA + 4% sucrose solution for 15 min. Pigment was removed from cells by photochemical bleaching in a bleaching solution (250 μL deionized formamide, 4 mL water, 250 μL of 20× Saline Sodium Citrate (SSC) (1× final), 817 μL 30% hydrogen peroxide) for 15 minutes under a stadium light (Yanycn, power: 400W LED, color: 5000k daylight white light), and then washed 5 times with 1×PBS. The reaction was then quenched with 50 mM NH_4_Cl for 10 minutes and washed 2–3 times with 1×PBS (62).

After bleaching, fixed cells were permeabilized with 0.25% TritonX-100 in 1×PBS for 10 minutes, blocked for 1 hour at RT, and then incubated with rabbit anti-FLAG 1:1,000 or mouse anti-Calnexin 1:200 primary antibodies for 1 hour at RT. Fixed cells were then incubated with anti-goat rabbit Alexa Fluor 647 1:1,000 (Invitrogen, Carlsbad, CA, #A-21446) and goat anti-mouse Alexa Fluor 594 1:1,000 (Invitrogen, Carlsbad, CA, #A-11005) secondary antibodies for 1 hours at RT. *Z* stacks were obtained using the Leica STELLARIS 8 FALCON Confocal (Leica, Wetzlar, Germany). Microscope and image analysis was performed using IMARIS 10.2 software (Oxford Instruments, Abingdon, England).

### Protein stability and cleavage studies.

ARPE-19 cells were seeded at 40,000 cells in 24-well tissue culture plates. After 24 hours, subconfluent cells were transduced with lentiviral vectors carrying WT-MYRF, C-terminal mutant MYRF, or V679A (MOI 3 from 10X concentrated stocks) vectors in the presence of 1 mg/mL LentiBoost. Cells were treated with 300 μg/mL cycloheximide (Sigma Aldrich, #C4859-1ML) and lysates collected in Pierce RIPA Buffer (Thermo Fisher Scientific, #89900) following 2 cell rinses in PBS at the following time points: 0, 12,16, 20, 24 hours. Protein concentration of cell lysates was quantified using Pierce BCA Protein Assay Kit (Thermo, #2325). Samples were denatured using 4× Laemmli Sample Buffer (Bio-Rad Laboratories, Hercules, CA) supplemented with 2-Mercaptoethanol and boiled at 95°C for 5 min. Upon SDS-page, samples were transferred onto Immobilon-FL PVDF membrane (Millipore Sigma, Burlington, MA) and probed chicken anti-GFP 1:5,000 (Abcam, Cambridge, England, #13970) or rabbit anti-GAPDH 1:1,000 (Millipore Sigma, #ABS16), as well as rabbit anti-FLAG 1:1,000 or mouse anti-MYRF^393-766^ (1) (gifted from Ben Emery, Oregon Health & Science University, Oregon, USA) primary antibodies. IRDye 800CW goat anti-rabbit IgG 1:20,000 (LICOR, Lincoln, NE, 926-32211) and IRDye 680RD donkey anti-chicken IgG 1:20,000 (LICOR, Lincoln, NE, #925-68075) were used as secondary antibodies. Blots were imaged using the Odyssey CLx and quantified using ImageStudio (LICOR).

### qPCR.

For ARPE-19 experiments, cells were seeded at 50,000 cells/well on 24-well plates. After 24 hours, cells were transduced with 1 × 10^–2^ dilution of WT-MYRF, C-terminal mutant MYRF, or V679A (10X concentrated) vectors in the presence of 100 mg/mL LentiBoost. After 72 hours, RNA was isolated from cells using the RNAqueous-Micro Total RNA Isolation Kit (Invitrogen, Carlsbad, CA) according to the manufacturer’s protocol. cDNA was generated using the SuperScript III system (Thermo Fisher Scientific, Waltham, MA) following the recommended protocol. qPCR was performed on the Applied Biosystems 7500 Real Time PCR System (Applied Biosystems, Foster City, CA) using the TaqMan Assay system with inventoried probes for *MYRF*, *TMEM98*, *B-ACTIN* (Thermo Fisher Scientific, Waltham,MA). For animal experiments, eye tissues were harvested from E15.5 or E18.5 mouse embryos or 8-month-old mice. The lens, cornea, and optic nerve were dissected, RNA was isolated and cDNA generated, and TaqMan Assays conducted with inventoried probes for Myrf and Tmem98 (Thermo Fisher Scientific, Waltham, MA). Critical cycle threshold levels of each sample were normalized to levels of β-actin for human cells and Hprt for animal experiments. Fold activity was calculated using the ΔΔCt method and reported relative to controls.

### In silico modeling.

PONDR (23) and AlphaFold2 (22) were used for in silico modeling of the WT-MYRF and dG-MYRF conserved C2 domain amino acid sequence. PONDR was used to predict differences in intrinsically ordered and disordered regions. VXLT combines 3 previous neural network models (VL1, XN, and XC) trained on NMR and x-ray crystallography data with varying lengths of disordered regions. VSL2 combines 2 predictors optimized for long (>30 residues) and short (≤30 residues) disordered regions (20, 21, 23, 65). Alphafold2 was used for de novo prediction of 3D structure of the C2 domain in the presence or absence of the C-terminal frameshift variant amino acid sequence.

### Generation of humanized Myrf alleles in mice.

Mice were genetically engineered using CRISPR-Cas9 to replace mouse *Myrf* exon 26 with the homologous human *MYRF* exon 27 and fused it with mouse exon 25, including the C-terminal coding region the human-3′UTR sequence (Supplemental Figure 3). This humanized *Myrf* allele (*Myrf^humWT^*) was generated by injecting fertilized eggs from C57BL/6J matings with a pair of spCas9 guide RNAs (25 ng/μL each) (gRNA, protospacer adjacent motif=NGG) that are 253 bps apart in outward orientation together with the spCas9 protein (NEB, 30 ng/μL). Sequences of the gRNAs are listed below: the upstream gRNA 5′-GCTCACCAGCAATGTCACC-3′ and the downstream gRNA 5′-GGCAGTGCCACAGCCGGGAC-3′. A single stranded oligo of 327 bps (IDT Megamer) carrying the human Exon27 sequence and homology arms of 100 bp each is also included in the injection mixture as the repair template for single strand DNA mediated homologous recombination (HR). The sequence of this HR template is 5′- TAGGACAAGGCTGCCCTGACCTCTGTCTTCCCTGTCTCTCTAGGGCACC TCTCATCAGTGGCCAGTAACCATCCTGTCCTTCCGTGAATTCACATACCACTTtCGGGTGACATTGCTGg GTCAGGCCAACTGCAGTTCAGAGGCTCTCGCCCAGCCAGCCACAGACTACCACTTCCACTTCTACCGCCTGTG TGACTGAGCTGCCCTCCTGACAGTGCCACAGCCGcGACTGGAGTCCCTGGGCCCTCAACACTGGATGCA AATGTGTTACACTGGAGCCTGCTGCAGGCCAGCTCTCTGCTCTTCACTGCTTCCCTTGACTGGGGA-3′ (327 bps). Founder mice were genotyped using primers (Forward: 5′-GTGG CTACCCTTGCTCTCAA-3′, Reverse: 5′-GGCGAGAGCCTCTGAACTG-3′).

We generated a matched *Myrf^humdG^* mouse that recapitulates the splice site mutation found in our previously described large familial cohort with nanophthalmos, starting with heterozygous Myrf^humWT/+^, and using the following guide RNA (5′-CACTTCCGGGTGACATTGCT-3′) and ssDNA repair template: 5′- TGGCCAGTAACCATCCTGTCCTTCCGTGAATTCACATACXCACTTCCGGGTGA CATTGCTGGTCAGGCCAACTGCAGTTCAGAGGCTCTCGCCCAG CCAGCCACAGACTACCACTTCCACT-3′. *RxCre;Myrf^fl/fl^* mice have been previously described (8). Mice were genotyped using standard methods (66) and primers listed above, followed by restriction digest with the BseYI restriction enzyme site to distinguish between the *Myrf^humdG^* and *Myrf^humWT^* alleles. Animals were also initially genotyped for *rd1*, *rd8*, and *rd10* according to established methods, to eliminate retinal degeneration alleles common in C57BL/6J mouse strains (67).

### RNAscope in situ hybridization.

Heads were harvested from E15.5 embryos. Samples were fixed in 4% buffered PFA (0.1M NaPO_4_, pH 7.3) overnight at 4°C, dehydrated through increasing concentrations of ethanol up to 70%. Samples were dehydrated, processed and sectioned as previously described (8, 10). RNAscope was performed using reagents from the RNAscope Multiplex Fluorescent Detect V2 kit (Advanced Cell Diagnostics [ACD], Newark, CA, #323110) as previously described (8, 10).

### Retinal layer measurements, whole eye measurements, and cell counting.

Eyes from 12-month animals were enucleated and aligned to capture optic nerve and central cornea in the same plane, and images were captured on the Leica MX10F dissecting microscope (Leica, Wetzlar, Germany). Ocular axial length was measured at 12 months using ImageJ (NIH) 1.51m9 software (68), by marking a line from the central cornea to the base of the optic nerve. For the analysis, the following number of animals/eyes were used: *Myrf^humWT/+^* control, *n* = 4 animals, 8 eyes; *Myrf^humdG/+^*, *n* = 5 animals, 9 eyes.

### Mouse ocular imaging and electrophysiology.

Eyes from 3-, 6-, 9-, and 12-month old control (*Myrf^humWT/+^*; *n* = 8 eyes, 4 mice) and heterozygous mutant mice (*Myrf^humdG/+^*; *n* = 9 eyes, 10 mice) were evaluated sequentially in vivo by fundus photography, spectral domain OCT, and ERG as previously described (8). Data from *Myrf^humdG/+^* and controls were evaluated for statistical significance with 1-way ANOVA in GraphPad Prism 10 (GraphPad Software, San Diego, CA, USA). If significant, subsequent pairwise comparisons were done with 2-tailed Student’s *t* test. A-wave and B-wave amplitudes from *Myrf^humdG/+^* eyes and control littermates were evaluated using 2-tailed Student’s *t* test in GraphPad Prism 10.

### RPE flat mounts.

Eyes were enucleated from 12-month mice, and cornea, lens, optic nerve, and retina tissues were removed. At least 3 animals/eyes per genotype/age were used. Optic cups were fixed, washed, and blocked as previously described (8). Primary antibody incubation occurred overnight at 4°C: TMEM98 (1:500, 14731-1-AP, Proteintech, Rosemont, IL), Phalloidin (1:400, #A12380, Thermo Fisher, Scientific, Waltham, MA). RPE samples were washed in PBS, incubated with Alexa-conjugated fluorescent secondary antibodies, and mounted as previously described (8). Images were taken on a Lecia DM6000 compound microscope.

### REShAPE RPE morphometric analysis.

RPE flat mounts (see above) were prepared from 12-month-old control (*Myrf^humWT/humWT^*; *n* = 4 eyes, 4 mice), heterozygous (*Myrf^humWT/humdG^*; *n* = 5 eyes, 10 mice) from a single cohort. RPE flat mounts were stained as above with phalloidin to outline RPE cell borders. They were imaged using the LASX Navigator tiling software on a Lecia DM6000 compound microscope at 20X. Tiled images were fed into the REShAPE software (27), processed, and analyzed by the REShAPE software to look at morphometrics such as cell size, aspect ratio, hexagonality, and number of neighboring cells. Data points for RPE morphometrics from each tile were combined per biological replicate. Control and heterozygous RPE morphometrics were analyzed by performing a Student’s *t* test on the median value.

### scRNA-seq analysis.

Eyes from E17.5 *Myrf^humWT/humWT^* (*n* = 3 mice, 6 eyes) and *Myrf^humdG/humdG^* (*n* = 3 mice, 6 eyes) were dissected removing the lens and optic nerve and leaving only the optic cup (retina/RPE/sclera/cornea) and dissociated as previously described (10). Eyes were processed for scRNA-seq at the University of Michigan Advanced Genomics Core as previously described using the CellRanger 7.1.0 pipeline (10, 69). SEURAT 3.1.2 package (70, 71) was used to performed integration, unsupervised clustering, and cell type identification on scRNA-seq of E17.5 optic cups from this study with previously published scRNA-seq datasets from E15.5 and P0 *RxCre;Myrf^fl/fl^* mouse optic cups and paired controls (10). Quality control thresholds were set to nFeature_RNA > 200 and percent.mt < 15. SEURAT 5.1.0 package was used to generate up- and downregulated differentially expressed genes in the RPE cluster. The ClusterProfiler 4.14.6 (72) package was used to look for enriched GO terms and GSEA (73). UCell was used to assign “scores” to enriched GO term and GSEA pathways in *Myrf^humWT/humWT^* (*n* = 3 mice, 6 eyes) and *Myrf^humdG/humdG^* mice on a per cell basis (74).

### Patient collection, sequencing, and analysis.

The patient cohort consisting of individuals with axial length < 21 mm or high hyperopia > +5.50 spherical equivalent was previously described (34). Clinical and biometric data were reviewed for patients. DNA from whole blood or saliva was extracted according to standard procedures as before. Patient samples were sequenced at the National Intramural Sequencing Center (Rockville, MD) by Illumina short-read WGS using the Illumina NovaSeq platform (Illumina, San Diego, CA). Reads were aligned and variants were called and annotated via a customized pipeline (https://github.com/Bin-Guan/NGS_genotype_calling; commit ID 32a0371, and https://github.com/Bin-Guan/variant_prioritization; commit ID 5cfb4f8).

### Minigene splicing assays and patient blood RNA analysis.

P04825 and P01965 WT and variant sequences were cloned into the pSLP3 exon trap vector. HEK293T (ATCC, #CRL-2316, American Type Culture Collection Manassas, VA) cells were seeded in 6-well plates at a density of 75,000 cells per well. Plasmid DNA (970 ng) and 30 ng GFP plasmid were transfected into HEK293T cells using FuGENE 6 transfection reagent (Promega, Madison, WI) at a 3:1 ratio. Cells were incubated overnight and then harvested the next day for RNA isolation using the QIAGEN Mini RNA Isolation Kit (Qiagen, Germantown, MD). cDNA was generated using SuperScript III and random primers following manufacturer’s protocols. Splicing within the exon trap vector was analyzed by amplifying across the exon trap with for V1 primer 5′-TCTGAGTCACCTGGACAACC-3′ and rev V2 primer 5′-ATCTCAGTGGTATTTGTGAGC-3′. PCR products were confirmed by Sanger sequencing. The PCR products were cloned into the TOPO TA cloning vector (Thermo Fisher Scientific, Waltham, MA). Eighteen colonies were picked and sequenced to test the frequency of splicing into the cryptic splice acceptor site.

Blood was collected from subject P04818 and stored in PAXgene tubes at –80°C until RNA preparation (Qiagen, Germantown, MD). Prior to isolating the RNA, the blood sample was equilibrated to RT for 2 hours. RNA was isolated using the protocol provided with the PAXgene Blood RNA Kit, PreAnalytix (Qiagen, Germantown, MD). RNA was treated with DNaseI (Invitrogen, Carlsbad, CA) and transcribed into cDNA using the High Capacity cDNA Reverse Transcription Kit (Applied Biosystems Foster City, CA). cDNA was amplified using a forward oligo (MYRF exon 23; 5′-ATCACCTCCCAGTACTGTGC-3′) and a reverse oligo (MYRF exon 25; 5′-GGTGTGGAGACTCTGTGGAA-3′) with standard PCR cycling and 55°C annealing. Normal splicing was represented by a 203 bp band and splicing including the pseudoexon was represented by a 324 bp band. A gel fragment corresponding to 150–350 bp was isolated from the amplified patient cDNA and control. DNA was isolated using the QIAEX gel extraction kit (Qiagen, Germantown, MD) and cloned into a TA vector using the TOPO TA Cloning for Sequencing Kit (Invitrogen, Carlsbad, CA). Twenty colonies were collected and analyzed by PCR and sequenced to calculate the occurrence of splicing into the cryptic splice site.

### Statistics.

A χ^2^ test was used to detect significant skewing of expected Mendelian ratios of genotypes at E12.5–P0. A Wilcoxon Rank Sum test was used to identify DEGs between 2 groups of cells in the scRNA-seq dataset. For DEG analysis, an *P*_adj_ value was calculated based on Bonferroni correction using all genes in the dataset. DEGs were considered significant with a *P*-adjusted value less than 0.1. One-way ANOVA was used when comparing the means of 3 or more groups. A 2-way ANOVA with multiple comparisons was used when comparing the means of 3 or more groups across genotypes and timepoints. A Student’s *t* test was used when comparing the means of only 2 groups. Unless otherwise noted, in the figure legend data represent mean ± SD.

### Study approval.

Patient work was approved by the IRB at the University of Michigan under protocol no. HUM00046159. All patients provided written informed consent for their inclusion. Mouse studies were approved by the IACUC at the University of Michigan under protocol nos. PRO00010115 and PRO00011748. This study was carried out in accordance with the Tenets of the Declaration of Helsinki.

### Data availability.

scRNA-seq data are available at the Gene Expression Omnibus (GEO, National Center for Biotechnology Information, National Library of Medicine; GSE310659). Genomic data are accessible at dbGAP (accession no. phs004462.v1.p1). The code used to process scRNA-sequencing data as well as analyze REShAPE morphometric and scRNA-sequencing differential gene expression has been uploaded as supplemental.txt files. Primary data are provided for all the experiments in the Supporting Data Values file. All additional relevant data can be found within the article, and its supplementary information and primary data are available upon request from the corresponding author.

## Author contributions

GMR and LP wrote the manuscript. GMR precedes MLB in co–first authors as she took the lead in the project and wrote the manuscript. GMR, MLB, and LP designed research studies, conducted experiments, acquired data, and analyzed data. SQW, CT, NTY, DK, RSL, and DS conducted experiments, acquired data, and analyzed data. LP, QZ, JYSH, JMLM, LD, SEM, JER, and RBH provided reagents. BG and DFH analyzed data.

## Funding support

This work is the result of NIH funding, in whole or in part, and is subject to the NIH Public Access Policy. Through acceptance of this federal funding, the NIH has been given a right to make the work publicly available in PubMed Central.

- NEI K08 EY032098 (to LP).
- E. Matilda Ziegler Foundation for the Blind (to LP).
- Bright Focus Foundation (M2022011N) (to LP).
- Research to Prevent Blindness Career Development Award (to LP).
- Knight Templar Eye Foundation funding (to LP).
- Vision Research Core (NEI P30-EY007003) (to LP).
- Shared Instrument Grant S10 (NIH SIG grant S10OD28612-01-A1) (to LP).
- NEI T32 EY013934 (to GMR).
- James Grosfeld Initiative for Dry AMD Trainee Grant (to GMR).
- NEI intramural funding (RBH, BG, and LD).
- NEI K08 - EY033420 (to JMLM).
- The James Grosfeld Initiative for Dry AMD (to JMLM.)
- Discovering Hope Foundation funding (to JMLM).
- NEI R01 EY011671 (to JER).
- BrightFocus Foundation Postdoctoral Fellowship M2024001F (to JYSH).
- Michigan Pioneer Fellows Program (to JYSH).
- Intramural Research Program of the National Institutes of Health (NIH), The National Eye Institute.

## Supplementary Material

Supplemental data

Supplemental data set 1

Supplemental data set 2

Unedited blot and gel images

Supporting data values

## Version 1 02/26/2026

In-Press Preview

## Version 2 03/23/2026

Electronic publication
