# Supplementary material for: Splicing variants in MYRF cause partial loss of function in the retinal pigment epithelium leading to nanophthalmos
Source: JCI Insight. 2026 Feb 26;11(6):e194681. doi: 10.1172/jci.insight.194681 (PMC13043084; doi:10.1172/jci.insight.194681)
Supplement: Supplemental data [file jciinsight-11-194681-s210.pdf]

SUPPLEMENTAL FIGURES

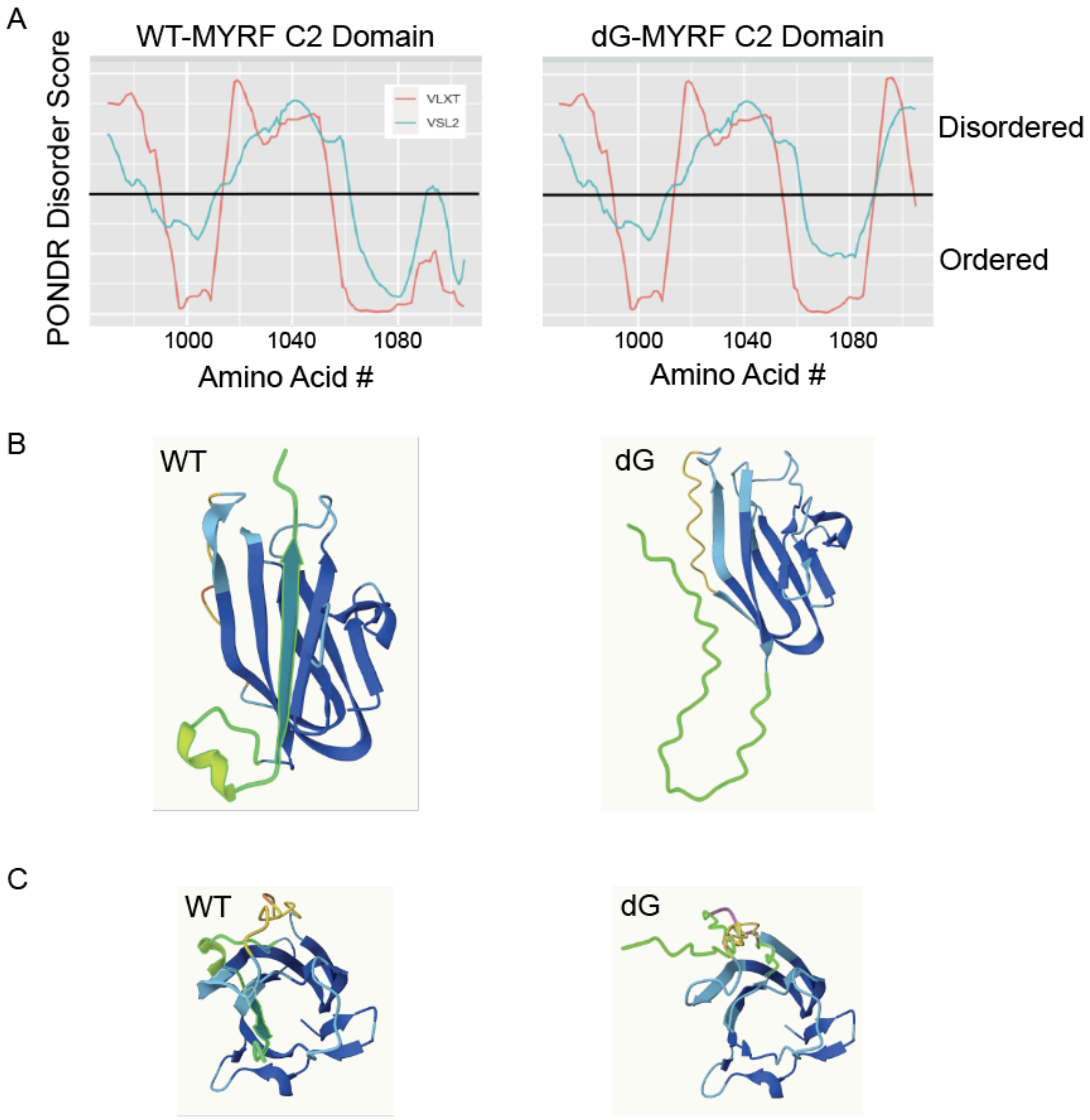

Supplemental Figure 1. In Silico Modeling of MYRF C-terminal Variant Protein. (A) The Predictor of Natural Disordered Regions (PONDR) algorithm suggests that the *dG-MYRF* variant creates a more disordered C2 domain than in *WT-MYRF* (PONDR score > 0.5 vs < 0.5, respectively). **(B-C)** AlphaFold2 de novo folding of the C-terminal domain of *MYRF* showing loss of beta sheet and alpha-helix structures as shown from the side **(B)** and from overhead **(C)**. Region highlighted in green is the amino acid sequence affected by the C-terminal frameshift variant.

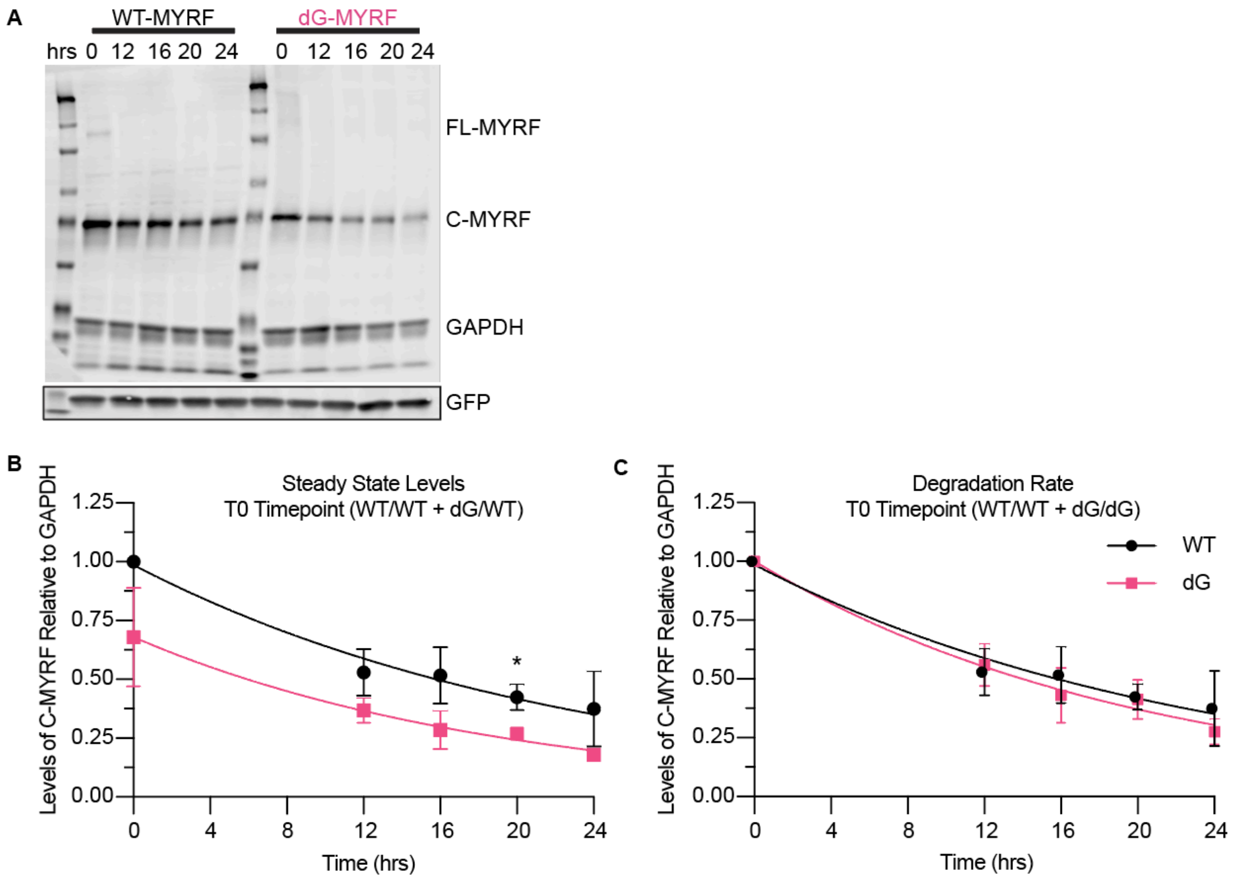

Supplemental Figure 2. Cycloheximide pulse-chase assay detects reduced steady state levels of variant dG-MYRF. (A-B) ARPE-19 cells were transduced with WT-MYRF and dG-MYRF. After 72 hours, cells were treated with 300ug/mL cycloheximide (CHX) to inhibit translation. Cell lysates were collected at 0, 12, 16, 20, and 24 hours. Steady state levels (0 hr timepoint) of the full length (~140kDa) or C-terminal fragment (~70kDa) of MYRF in the dG-MYRF protein were decreased compared to WT-MYRF by Western blotting **(A)** and quantified relative to GAPDH (*p<0.05) **(B)**. **(C)** After normalizing to time =0, there was no difference in the degradation rates of WT, dG-MYRF full length (~140kDa) or C-terminal fragments (~70kDa) over a 24-hour chase period (n=3).

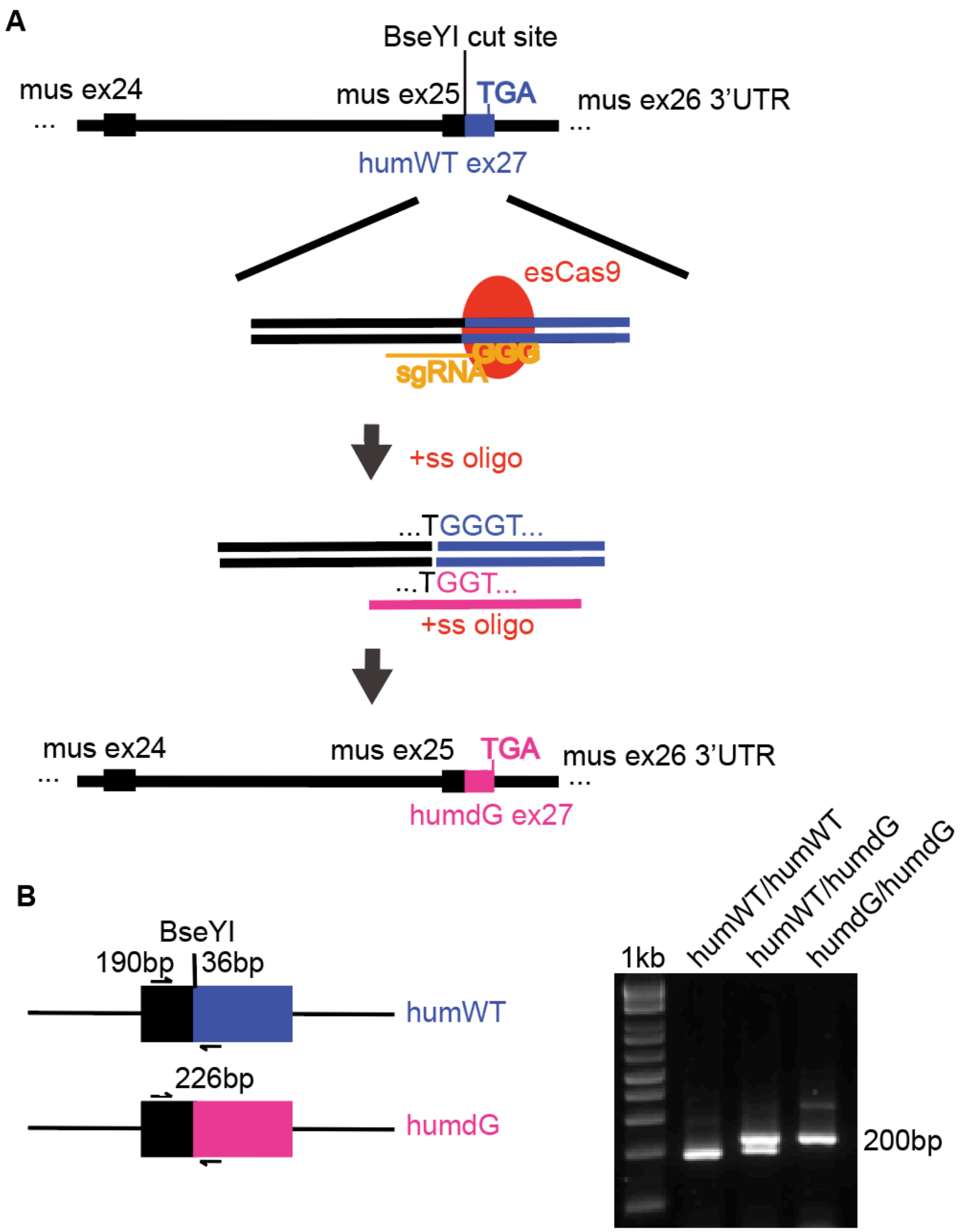

Supplement Figure 3. Generation of the humanized C-terminal *Myrf* mouse model. **(A)** CRISPR/Cas9 strategy to generate the humanized WT (humWT) and the C-terminal variant *Myrf* (humdG) alleles. **(B)** Genotyping strategy to differentiate humWT vs. humdG alleles using the BseY1 cut site which is lost in the humdG sequence.

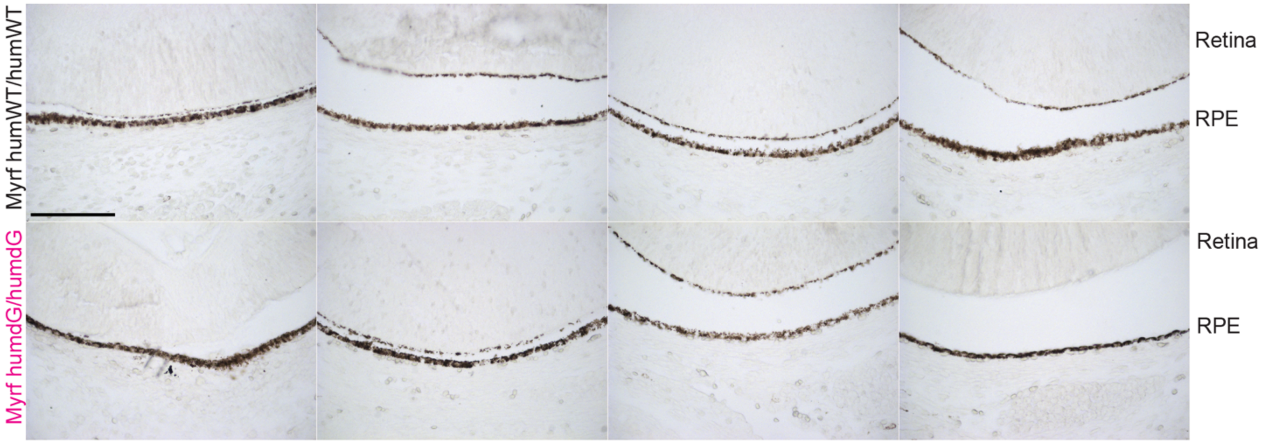

Supplemental Figure 4. *Myrf*$^{humdG/humdG}$ show no signs of depigmentation at embryonic stages. Brightfield images from E15.5-E16.5 embryos showing no change in pigmentation in the RPE layer of homozygous *Myrf*^{humdG} embryos relative to *Myrf*^{humWT} (scale bar = 100µm) (n=6-8 embryos per genotype).

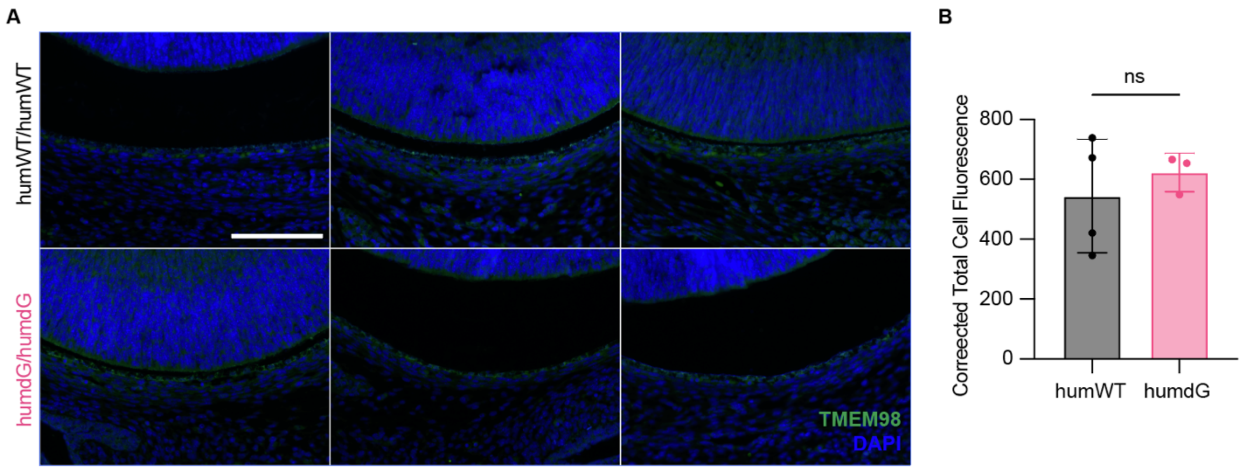

Supplemental Figure 5. ***Myrf*$^{humdG/humdG}$ show no reduction in TMEM98 protein at embryonic stages. (A)** IHC images of TMEM98 staining in E15.5 embryos showing no change immunoreactivity in the RPE layer of homozygous *Myrf*^{humdG} embryos (scale bar = 100μm) (n=3-4 embryos per genotype). **(B)** Quantification of TMEM98 protein staining by corrected total cell fluorescence by Student's T-test.

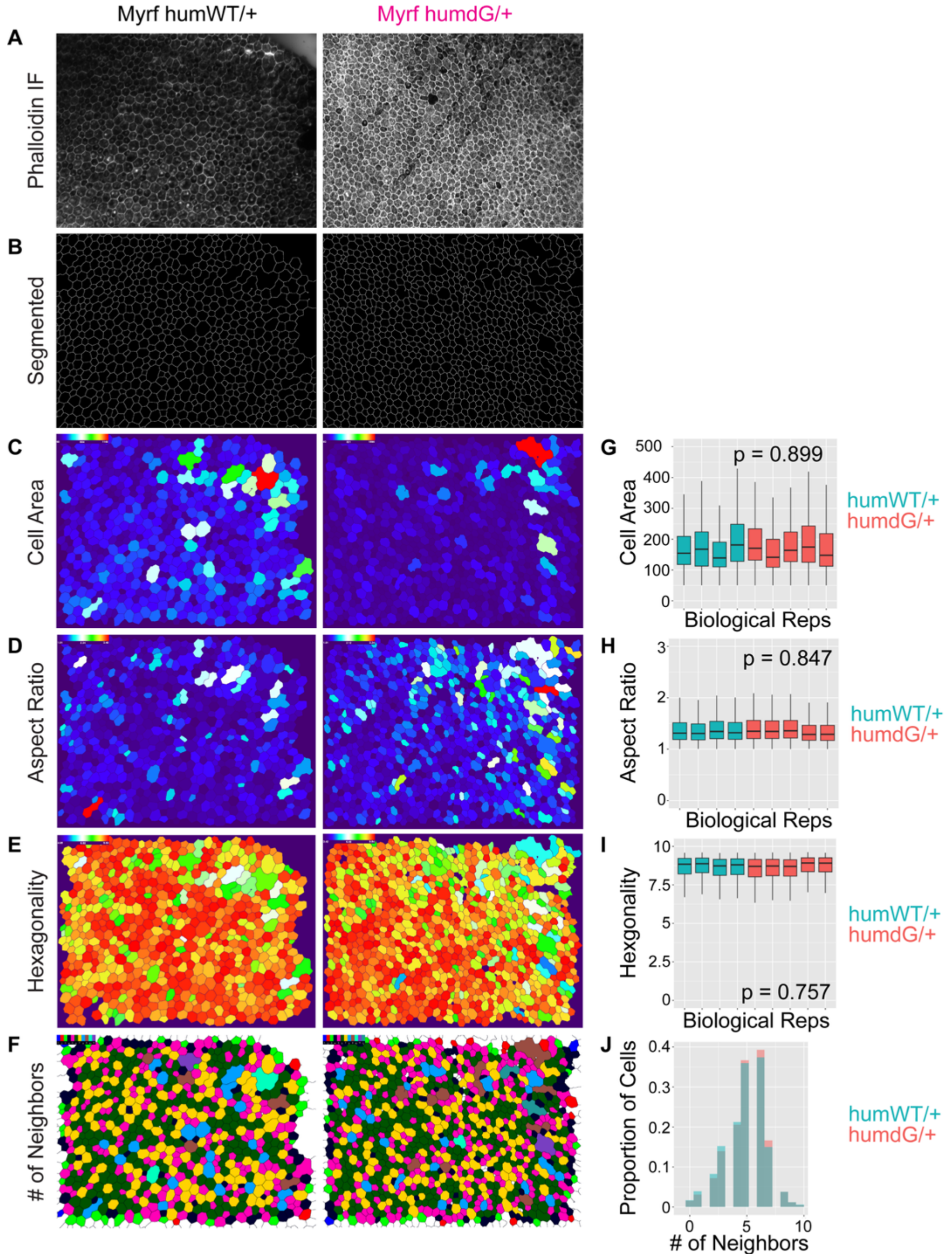

Supplemental Figure 6. Morphometric analysis of *Myrf*$^{humdG/+}$ RPE reveals no differences in morphometrics. (A-F) Representative output images from modified REShAPE AI software analysis examining segmentation **(B)**, cell area **(C)**, aspect ratio **(D)**, hexagonality **(E)**, # of neighbors **(F)** starting from an RPE flat mount stained with rabbit anti-phalloidin (1:400) to outline cell borders (n=4-5 per genotype). **(G-J)** Student's t-test showed no significant differences when comparing median values for cell size **(G)**, aspect ratio **(H)**, and hexagonality **(I)**, and number of neighbors between genotypes. Performed on 20X images.

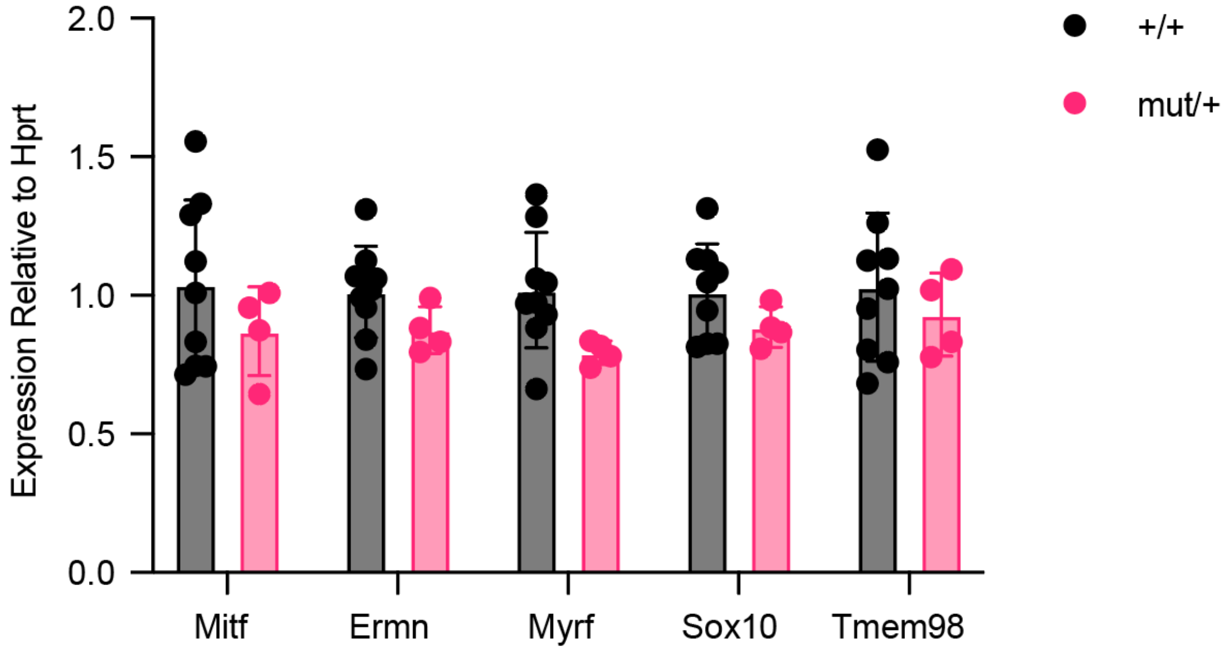

Supplemental Figure 7. No Change in Expression of Key RPE Genes in Myrf$^{humdG/+}$ mice. Expression of key RPE genes in P21 Myrf$^{humdG/+}$ vs. Myrf$^{+/+}$ mouse optic cups (n=4-9 pairs of optic cups per genotype). No significant difference in expression of select RPE genes are seen in P21 optic cups by Student's T-test.

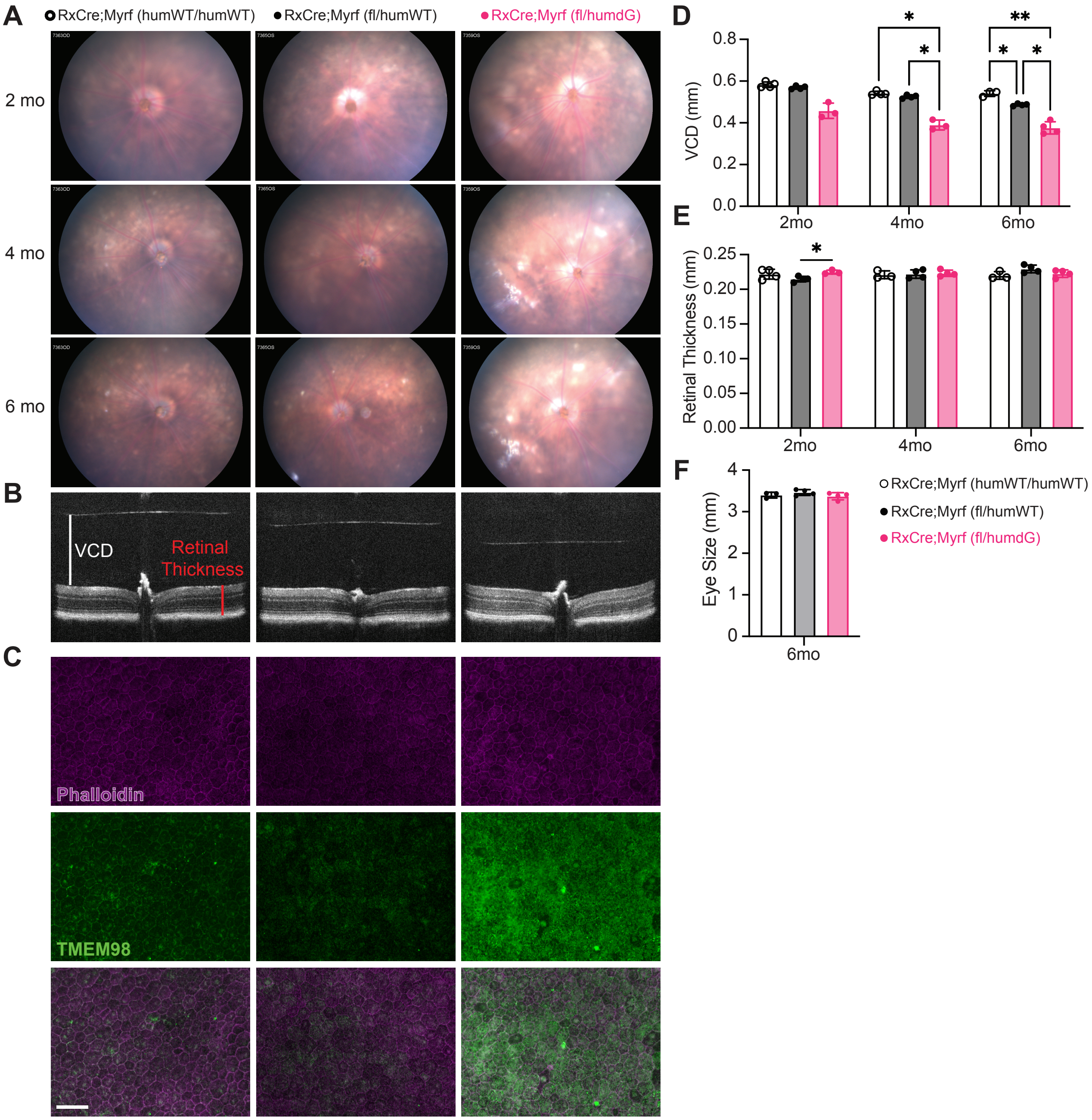

Supplemental Figure 8. Humanized C-terminal allele reduces VCD and causes RPE degeneration in vivo. (A) Fundus photos from 2, 4, and 6-month-old mice with the following genotypes: RxCre;Myrf$^{humWT/humWT}$, *RxCre;Myrf*$^{fl/humWT}$, or *RxCre;Myrf*$^{fl/humdG}$ (n=4 eyes/2 mice, per geno) demonstrating significant RPE degeneration in the presence of the humdG allele. **(B)** OCT images of *RxCre;Myrf*$^{fl/humdG}$ and respective controls showing reduced vitreous chamber depth (VCD, white bar) at 6 months and no difference in overall retinal thickness (red bar). **(C)** Representative images of immunofluorescent staining of TMEM98 (green) and phalloidin (red) in RPE flat mounts from 6-month-old *RxCre;Myrf*$^{fl/humdG}$ and respective controls (n=2 mice per genotype) showing mislocalization of TMEM98 in *RxCre;Myrf*$^{fl/humdG}$ mice compared to controls. Scale bar = 50µm. **(D)** Quantification of the VCD (mm) at 2, 4, and 6-months showing significant reduction of VCD in the presence of the humdG allele at the 4 and 6 months. **(E)** Quantification of the total retinal thickness (mm) at 2, 4, 6-months showing comparable retinal thickness across each genotype over time. Mean ± standard deviation. **, $p < 0.01$; *, $p < 0.05$ by Two-way ANOVA (**D** and **E**). (**F**) Quantification of the eye size (mm) at 6-month timepoint after dissection.

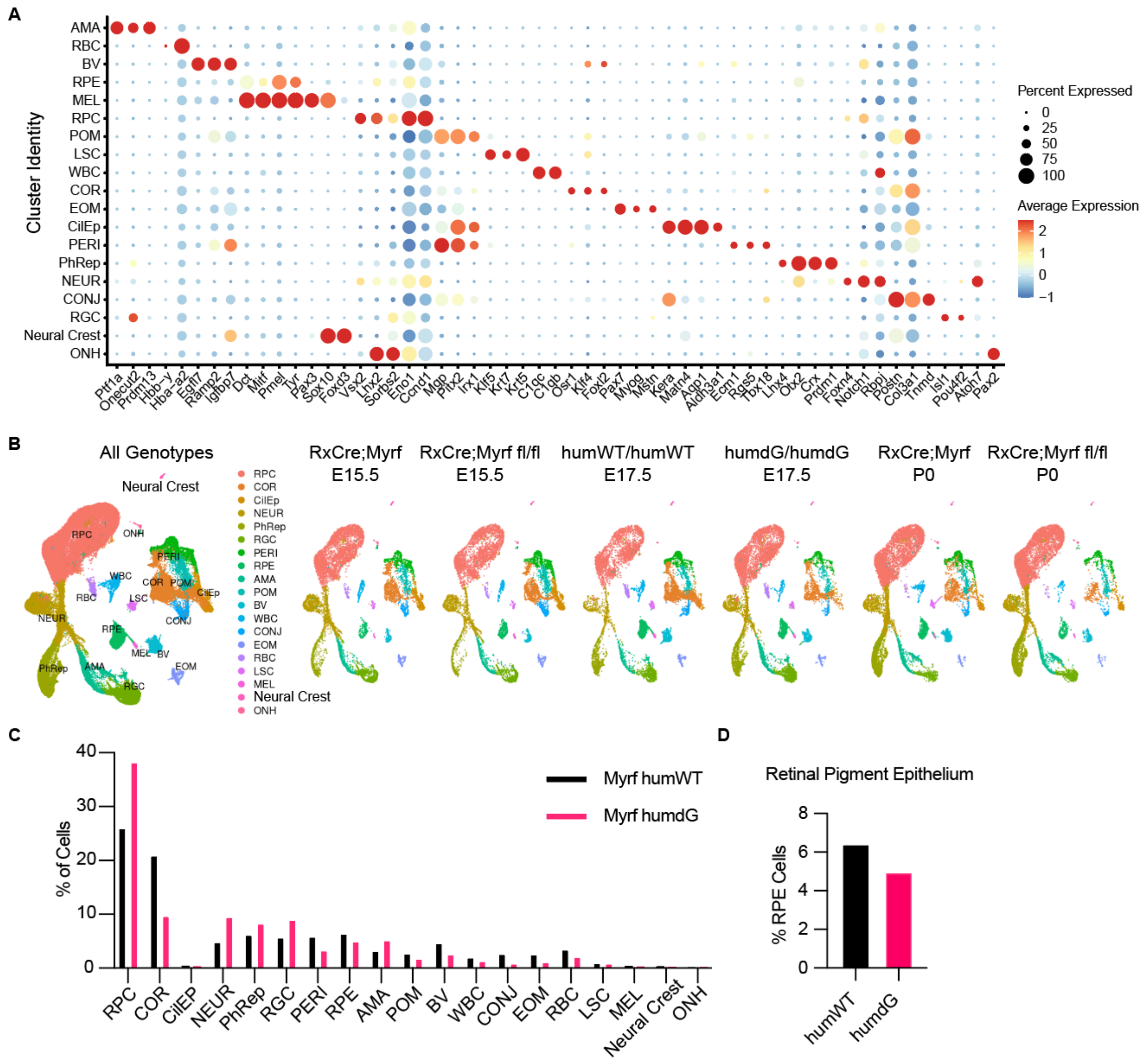

Supplemental Figure 9. Identification of Optic Cup Clusters in Single Cell RNA-sequencing Dataset. (A) Markers previously established in the field (12) were used for the identification of all major cell types within the optic cup. **(B)** Previously published *RxCre;Myrf*$^{fl/fl}$ E15 and P0 datasets (12) were integrated with the humanized *Myrf* datasets to improve clustering and used for cross comparison of downstream analyses. All datasets contained all major cell types, and no novel clusters were observed. **(C-D)** Percent of cells in each cluster by genotype **(C)**. Percent of RPE cells in humWT (5%) compared to humdG optic cup (6.4%) **(D)**.

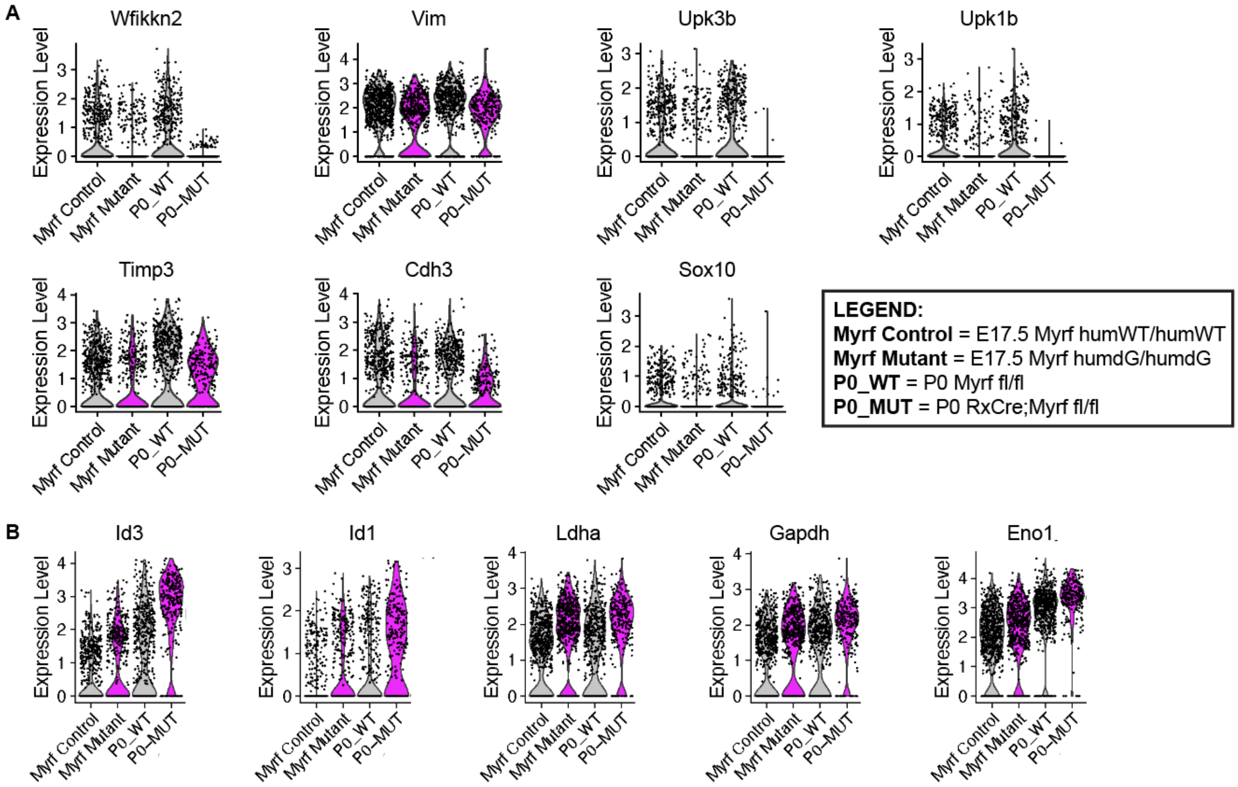

Supplemental Figure 10. Concordance of Shared DEGs in Humanized C-Terminal Variant and Conditional Knock Out *Myrf* Mouse Models. Downstream analysis showing violin plots, with single cells plotted for selected differentially expressed genes in both $Myrf^{humdG/humdG}$ and $RxCre;Myrf^{fl/fl}$ mice was performed. Shared downregulated **(A)** and upregulated **(B)** genes related to RPE development and maintenance were all similarly altered in $Myrf^{humdG/humdG}$ and $RxCre;Myrf^{fl/fl}$ mice relative to their controls, but the magnitude of the effect was greater for some genes in $RxCre;Myrf^{fl/fl}$ relative to $Myrf^{humdG/humdG}$. For example, note *Id3* and *Id1.*

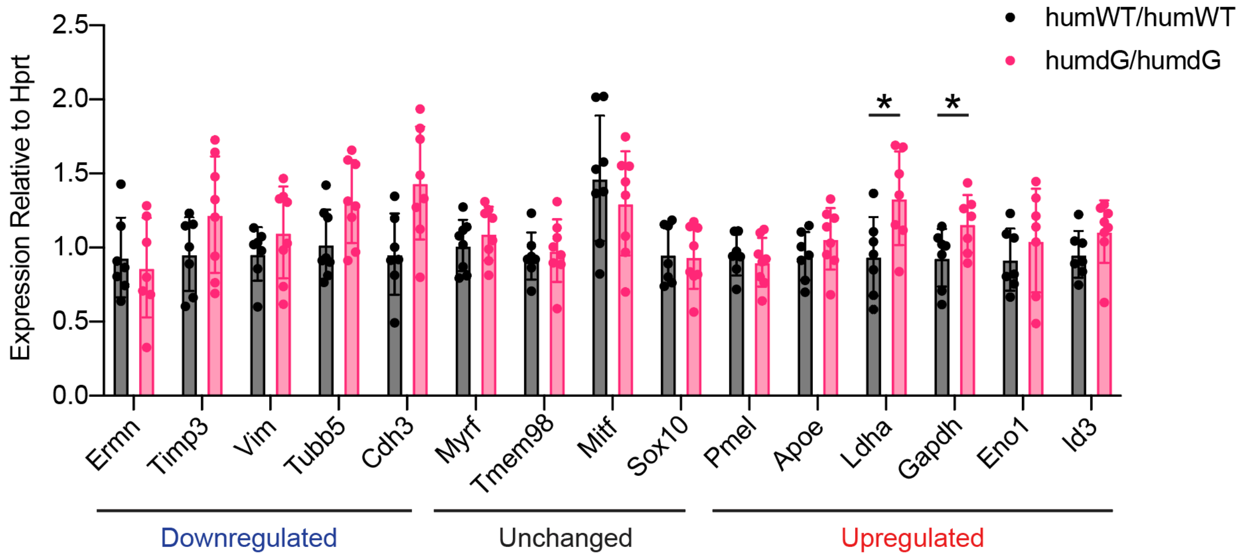

Supplemental Figure 11. Validation of Unchanged and Upregulated scRNA-seq Hits via RT-qPCR in E16.5 Whole Eyes. Expression of downregulated, unchanged, or upregulated differentially-expressed genes from scRNA-seq dataset in whole eyes of E16.5 *Myrf*$^{humWT/humWT}$ and *Myrf*$^{humdG/humdG}$ embryos. Unchanged hits *Myrf, Tmem98, Mitf*, *Sox10* show no difference in expression between genotypes in E16.5 whole eyes via RT-qPCR. Upregulated hits *Ldha* and *Gapdh* show significant upregulation in expression in *Myrf*$^{humdG/humdG}$ E16.5 whole eyes via RT-qPCR. Downregulated hits showed no significant changes in expression between genotypes in E16.5 whole eyes. Outliers were identified by Grubbs test (Alpha = 0.2) and removed. *, $p < 0.05$ by Student's T-test.

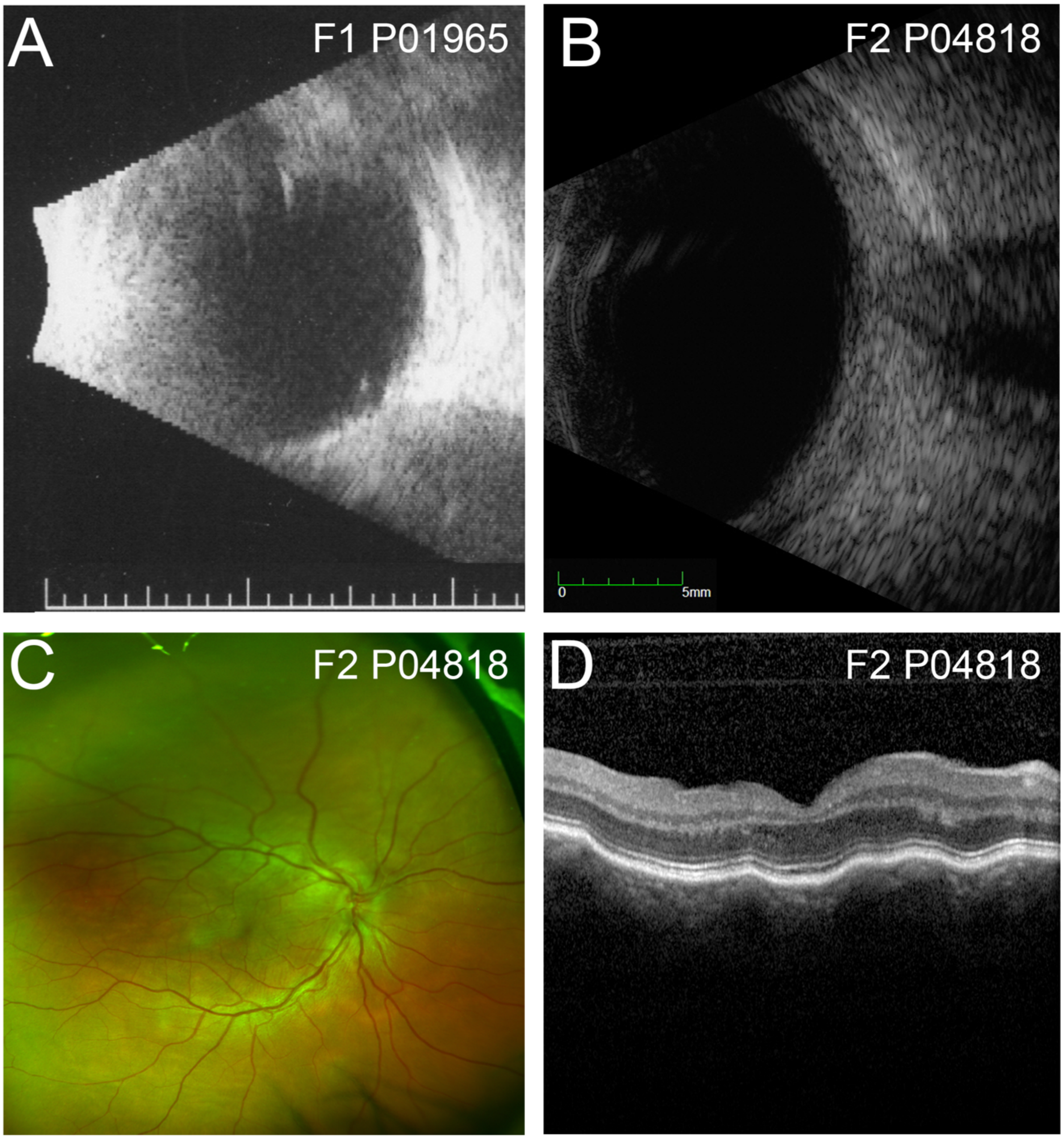

Supplemental Figure 12. Clinical features of families carrying MYRF intronic variants. (A-B) Ultrasound of right eyes of P01965 **(A)** and P04818 showing increased scleral thickness and reduced axial length 17.4 mm and 16.9 mm, respectively. **(C)** Optos photo of P04818 right eye showing vascular tortuousity, optic disc crowding, and no signs of retinal degeneration. **(D)** SD-OCT of P04818 showing choroidal folds.

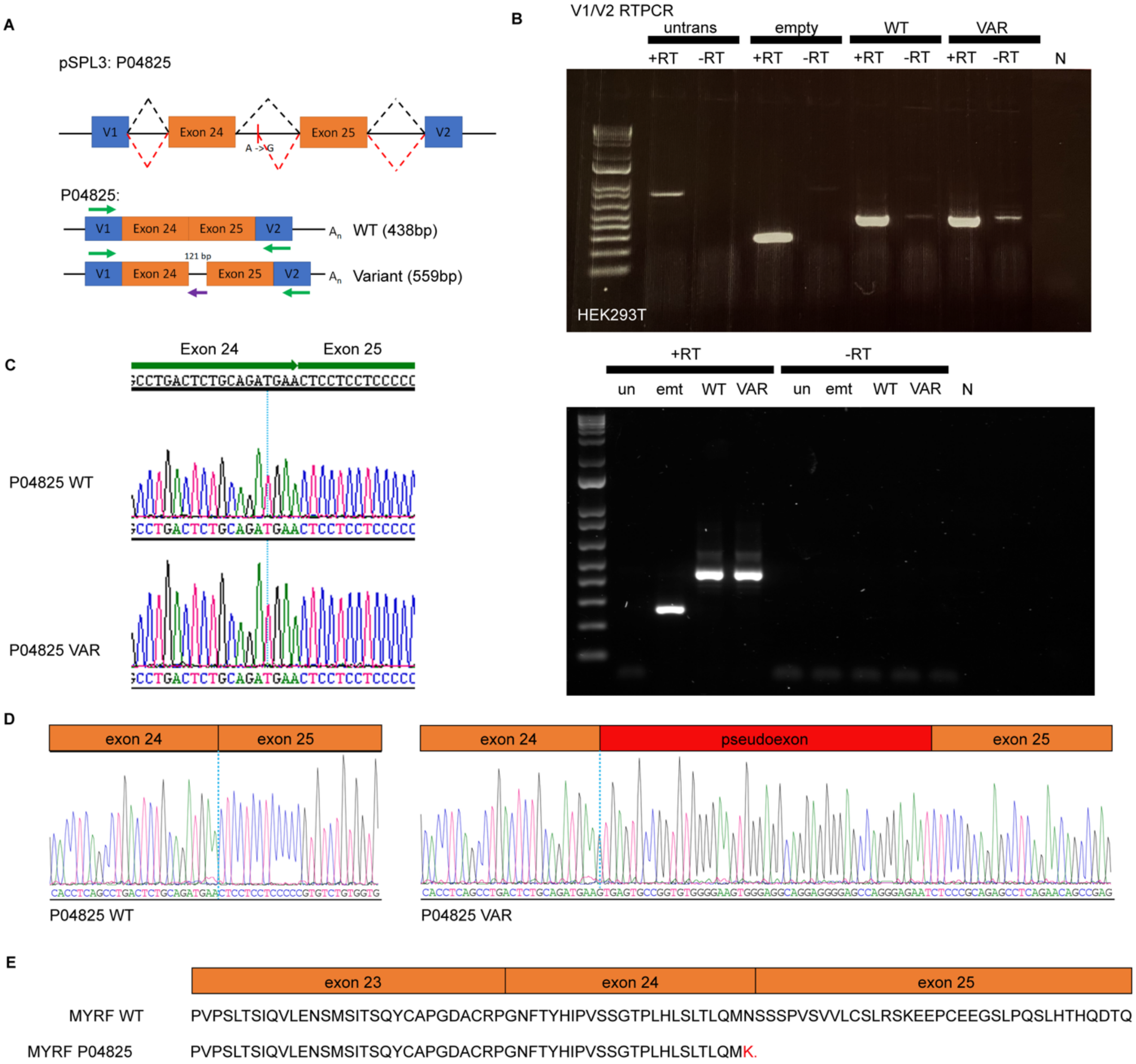

Supplemental Figure 13. Predicted Splice Variant P04825 does not use alternative splice site in minigene assays. Schematic of mini gene assay performed to determine effects of intronic variant on splicing and expected results **(A)**. Variant c.3194+122A>G is spliced properly in HEK293T or ARPE-19 cells **(B)**. Lanes included untransfected cells (un or untrans), empty vector, wild type (WT) MYRF exon 24 and 25, and the c.3194+122A>6 variant (VAR). Chromatograms from DNA sequencing of the PCR products only detected normal splice site usage in mutants **(C)**. Sequencing from TA-cloned PCR products of cDNA amplified from patient blood for variant c.3194+122A>G shows altered splicing and inclusion of a pseudoexon **(D).** Translation of sequenced cDNA reveals that altered splicing induces a stop codon leading to early truncation of the MYRF after exon 24 **(E).**

Supplementary Table 1: Shared DEGs Between MYRFhumMUT and P0 conditional KO

Gene	Humanized Mouse Model (Myrf hWT/hMUT)					Conditional KO Model (RxCre;Myrf fl/fl)				
	p_val.x	HumFC	pct.1.x	pct.2.x	p_val_adj.x	p_val.y	P0_FC	pct.1.y	pct.2.y	p_val_adj.y
Padi2	8.61E-09	-2.4290381	0.038	0.132	2.10E-04	8.68E-08	-1.9211933	0.016	0.138	0.00211807
Ppp1r16b	2.99E-17	-2.184952	0.067	0.248	7.30E-13	3.49E-10	-4.4825747	0.012	0.164	8.52E-06
A930028N01	5.68E-12	-1.6914712	0.12	0.274	1.39E-07	8.57E-25	-6.477241	0.004	0.337	2.09E-20
Rasgrp3	7.43E-20	-1.5360456	0.195	0.427	1.81E-15	6.67E-27	-5.7056312	0.039	0.401	1.63E-22
Col5a2	9.16E-26	-1.3483427	0.172	0.476	2.24E-21	4.96E-10	-3.1773161	0.181	0.361	1.21E-05
Tmem229b	4.67E-11	-1.3379554	0.134	0.285	1.14E-06	1.51E-08	-3.3814417	0.094	0.25	3.69E-04
Col3a1	4.43E-52	-1.2899615	0.538	0.939	1.08E-47	7.13E-37	-4.6805495	0.157	0.603	1.74E-32
Dcn	1.19E-28	-1.2454641	0.258	0.588	2.91E-24	6.80E-19	-2.6825776	0.217	0.514	1.66E-14
Wfikkn2	5.61E-09	-1.1103496	0.212	0.359	1.37E-04	2.17E-19	-4.5431578	0.154	0.424	5.30E-15
Col1a1	4.23E-48	-1.108001	0.746	0.977	1.03E-43	1.04E-68	-4.2314607	0.287	0.9	2.54E-64
Col5a1	1.69E-16	-1.1008552	0.162	0.386	4.11E-12	1.14E-11	-4.1803179	0.146	0.342	2.78E-07
Col1a2	2.14E-46	-1.0730269	0.697	0.976	5.22E-42	8.60E-56	-3.2611329	0.555	0.923	2.10E-51
Cdh3	1.10E-10	-1.0686517	0.309	0.479	2.69E-06	1.04E-06	-1.4447087	0.547	0.534	0.02533827
Ramp2	6.20E-10	-1.0468211	0.177	0.328	1.51E-05	1.70E-36	-5.2070951	0.031	0.492	4.16E-32
Fbln2	2.51E-09	-1.0078024	0.149	0.295	6.13E-05	1.60E-13	-3.4955629	0.087	0.31	3.90E-09
Upk1b	1.65E-09	-0.982261	0.135	0.275	4.02E-05	1.07E-24	-5.8897906	0.016	0.352	2.62E-20
Col6a3	8.51E-20	-0.9553488	0.143	0.413	2.08E-15	3.00E-11	-3.0709538	0.031	0.215	7.33E-07
Col14a1	2.10E-16	-0.9100164	0.139	0.375	5.14E-12	3.33E-08	-5.074051	0.02	0.148	8.13E-04
Upk3b	7.60E-09	-0.8336197	0.231	0.382	1.86E-04	8.86E-33	-6.3087168	0.012	0.432	2.16E-28
Col12a1	1.77E-10	-0.813864	0.103	0.258	4.31E-06	4.63E-14	-6.6053469	0.016	0.222	1.13E-09
Gucy1a1	3.98E-08	-0.7656024	0.156	0.292	9.71E-04	4.02E-07	-2.6182813	0.118	0.262	0.00982239
Lum	7.71E-09	-0.6763242	0.141	0.289	1.88E-04	5.24E-25	-6.1675238	0.024	0.366	1.28E-20
Sox10	2.51E-06	-0.6366696	0.156	0.269	0.06130799	1.51E-15	-2.7631164	0.035	0.275	3.69E-11
Vim	4.83E-18	-0.6033612	0.655	0.923	1.18E-13	1.37E-08	-0.5002305	0.878	0.84	3.35E-04
Gm42418	4.43E-27	-0.4386939	1	1	1.08E-22	1.25E-29	-0.8836091	1	1	3.04E-25
Timp3	5.68E-07	-0.4129193	0.342	0.513	0.01385337	1.55E-06	-1.085068	0.689	0.638	0.03771103
Cpe	2.71E-07	-0.352342	0.33	0.511	0.00662045	1.23E-09	-2.2066684	0.299	0.444	3.01E-05
Hist1h4h	1.48E-59	2.37574774	0.693	0.301	3.62E-55	7.73E-07	0.52701568	0.236	0.1	0.01887067
Hist1h2ac	2.51E-07	2.06143437	0.12	0.043	0.00611402	1.29E-08	1.1057587	0.193	0.06	3.15E-04
Nnat	3.55E-26	1.77823449	0.557	0.323	8.66E-22	7.42E-07	0.36941756	0.461	0.251	0.01811645
Hist1h1d	9.09E-20	1.5992939	0.496	0.295	2.22E-15	2.41E-07	0.94317392	0.154	0.046	0.00588459
Id1	2.38E-09	1.24813453	0.365	0.236	5.82E-05	2.70E-23	1.38557221	0.701	0.332	6.60E-19
Id3	2.37E-09	1.09360655	0.5	0.419	5.78E-05	5.48E-44	1.59423662	0.894	0.607	1.34E-39
Csrp2	2.21E-06	1.03813275	0.416	0.332	0.05404357	4.56E-22	1.1569519	0.795	0.488	1.11E-17
Ldha	2.76E-30	0.91195372	0.828	0.718	6.74E-26	4.30E-21	0.83002459	0.878	0.679	1.05E-16
Igfbp2	2.20E-12	0.85547762	0.622	0.5	5.38E-08	4.42E-23	1.33520614	0.87	0.719	1.08E-18
Pgam1	1.99E-14	0.73640801	0.695	0.615	4.86E-10	3.93E-10	0.41017261	0.898	0.725	9.60E-06
Tpi1	6.69E-13	0.67554877	0.716	0.645	1.63E-08	4.16E-11	0.54673703	0.894	0.703	1.01E-06
Pkm	6.61E-16	0.63555949	0.796	0.722	1.61E-11	1.74E-16	0.5262821	0.961	0.787	4.25E-12
Eno1	1.29E-10	0.59223741	0.697	0.635	3.15E-06	1.73E-15	0.59974977	0.906	0.707	4.22E-11
Pgk1	1.31E-06	0.53854368	0.662	0.614	0.03194289	5.71E-25	1.02859633	0.898	0.641	1.39E-20
Rps29	1.36E-30	0.52578933	0.996	0.997	3.33E-26	3.31E-11	0.2963416	0.996	0.98	8.07E-07
Rps13	4.86E-16	0.48302699	0.96	0.947	1.19E-11	3.18E-09	0.29802274	0.984	0.927	7.77E-05
Rps10	6.03E-19	0.48209411	0.973	0.973	1.47E-14	1.98E-08	0.26850535	0.984	0.953	4.84E-04
Rpl15	2.81E-15	0.46655307	0.935	0.916	6.85E-11	7.44E-11	0.31861905	0.949	0.914	1.82E-06
Rps28	5.94E-17	0.45964916	0.954	0.957	1.45E-12	4.32E-08	0.27782396	0.941	0.913	0.00105487
Atp5g2	1.15E-06	0.45963404	0.687	0.652	0.02817153	1.02E-13	0.46302427	0.909	0.78	2.49E-09
Ppia	1.22E-19	0.45182984	0.983	0.96	2.98E-15	5.28E-12	0.31265461	1	0.982	1.29E-07
Gapdh	6.77E-11	0.44384789	0.891	0.859	1.65E-06	3.61E-23	0.55224595	0.98	0.936	8.80E-19
Rps18	6.93E-17	0.43336289	0.96	0.957	1.69E-12	4.06E-10	0.27620586	0.984	0.956	9.91E-06
Eef1b2	2.87E-06	0.41823839	0.8	0.811	0.06994704	1.13E-07	0.33853738	0.925	0.821	0.00275352
Tuba1b	1.22E-06	0.40039428	0.731	0.751	0.0298906	2.05E-07	0.41470986	0.921	0.831	0.0050137
Rps24	8.90E-18	0.39327435	0.998	0.996	2.17E-13	2.10E-08	0.27256875	0.988	0.967	5.12E-04
Rpl29	9.54E-08	0.39008114	0.847	0.858	0.00232914	1.22E-08	0.29023579	0.961	0.883	2.98E-04
Fau	2.76E-13	0.36776227	0.981	0.977	6.74E-09	4.03E-11	0.27661819	0.984	0.949	9.84E-07
Rpl41	1.17E-12	0.34505763	0.969	0.983	2.85E-08	1.13E-28	0.49267313	0.996	0.974	2.77E-24
Rps7	8.81E-10	0.33816186	0.968	0.962	2.15E-05	8.48E-07	0.27563809	0.988	0.918	0.02069986
Rpl11	1.37E-09	0.33690149	0.968	0.949	3.34E-05	1.15E-12	0.32537874	0.988	0.938	2.80E-08
Rpl3	3.59E-08	0.32687341	0.895	0.92	8.76E-04	2.51E-06	0.26231742	0.957	0.911	0.06115496
Rpl23	1.81E-13	0.32420861	0.989	0.996	4.42E-09	8.75E-10	0.2918244	0.992	0.96	2.14E-05
Rpl19	4.75E-08	0.29635087	0.947	0.952	0.00115988	4.59E-11	0.30327259	0.992	0.936	1.12E-06
Rps19	1.61E-06	0.27159438	0.971	0.981	0.03935115	1.84E-07	0.27631867	0.976	0.944	0.00448985
Rpl26	3.61E-06	0.27092542	0.95	0.949	0.08816051	7.76E-11	0.29954988	0.969	0.923	1.89E-06

Gene had to have avg_log2FC >= abs(0.25) for BOTH genotypes

Gene had to have p_adj_val <= 0.1 for BOTH genotypes

Supplemental Table 2. Clinical features of MYRF nanophthalmos families.

Patient #	Family #	Sex	Ethnicity	Age at Exam	Sporadic/Familial	Glaucoma	Narrow angle?	Retinal Folds	Choroidal Folds	Pigmentary Retinopathy	Complications
P04818	F2	F	EUWA	53	Familial	+	+	-	+	-	Aqueous misdirection
P04825	F2	F	EUWA	75	Familial	NR	NR	NR	NR	NR	NR
P01965	F1	F	EUWA	60	Sporadic	+	+	NR	NR	+	RDD, phthis is OS

logMAR		Phakic Refraction (SE)		Lens Status		Axial Length (mm)		Phakic ACD		C/D Ratio		Max Clinic IOP		Scleral Thickness	
OD	OS	OD	OS	OD	OS	OD	OS	OD	OS	OD	OS	OD	OS	OD	OS
0.0969	0.301	NR	NR	Pseudo	Pseudo	16.93	16.75	2.75	2.39	0.15	0.1	16.5	16	1.9	1.8
NR	NR	NR	NR	NR	NR	18.77	18.67	NR	NR	NR	NR	NR	NR	NR	NR
0.3979	NLP	7.25	7.25	Pseudo	Pros	17.4	17.1	NR	NR	0.99	ND	38	40	+thick	+thick

RDD, rheumatogenous retinal detachment; Pseudo, pseudophakia; Pros, prosthesis; NLP, no light perception, NR, not recorded; C/D, cup to disc ratio; ACD, anterior chamber depth; SE, spherical equivalent; MAR, minimal angle of resolution; IOP, intraocular pressure; EUWA, European or White American.

Supplemental Table 3. Splicing variants in silico analysis.

Family	Gene	Position (hg19)	cDNA change	SpliceAI score	Pangolin score	Expected Effect	
F1	*MYRF*	Chr11:61536960 G>A	c.460+167G>A	0.992	0.78	Acceptor Gain	Intron 4 pseudoexon insertion causing frameshift
F2	*MYRF*	Chr11:61551519 A>G	c.3194+122A>G	0.8	0.49	Donor Gain	Gain cryptic donor exon 25 or exon 24 skipping

Supplemental Table 4. AMD Risk SNP Genotyping Primers

Gene	Primer (5' > 3')
CFH_F	ATTTCTTTTTGTGCAAACCTTTGTT
CFH_R	AACAAGGTGACATAAACATTTTGCC
C3_F	GTGGTTGACGGTGAAGATCC
C3_R	ATAATGGGCAGGCAAGGAGG
AH_F	TGGCAGCATGTTCTAAATGTG
AH_R	TAAAAGCCAACCCCAGGC
C2_F	CTAGTGTCTCCCCTGGTCC
C2_R	CCTGAGAGGGTCCATCTTCT

Supplemental Table 5. Quik Change Mutagenesis Primers

**Primer Name**	**Primer (5' > 3')**
dG-MYRF_F	GCACTGCTGGTCAGGCCAACTGCAGTTCAGAGG
dG-MYRF_R	TGGCCTGACCAGCAGTGCCACCCGAAAGTGGTA
V679A_F	GAGAACGCAGGGGCCGTGAAGGAGCTGTGCAAG
V679A_R	CACGGCCCCTGCGTTCTCCATGAAGATCCTTTC
