# Supplementary figures and images for: Splicing variants in MYRF cause partial loss of function in the retinal pigment epithelium leading to nanophthalmos
Source: JCI Insight. 2026 Feb 26;11(6):e194681. doi: 10.1172/jci.insight.194681 (PMC13043084; doi:10.1172/jci.insight.194681)

# Full unedited blot/gel for Figure 1

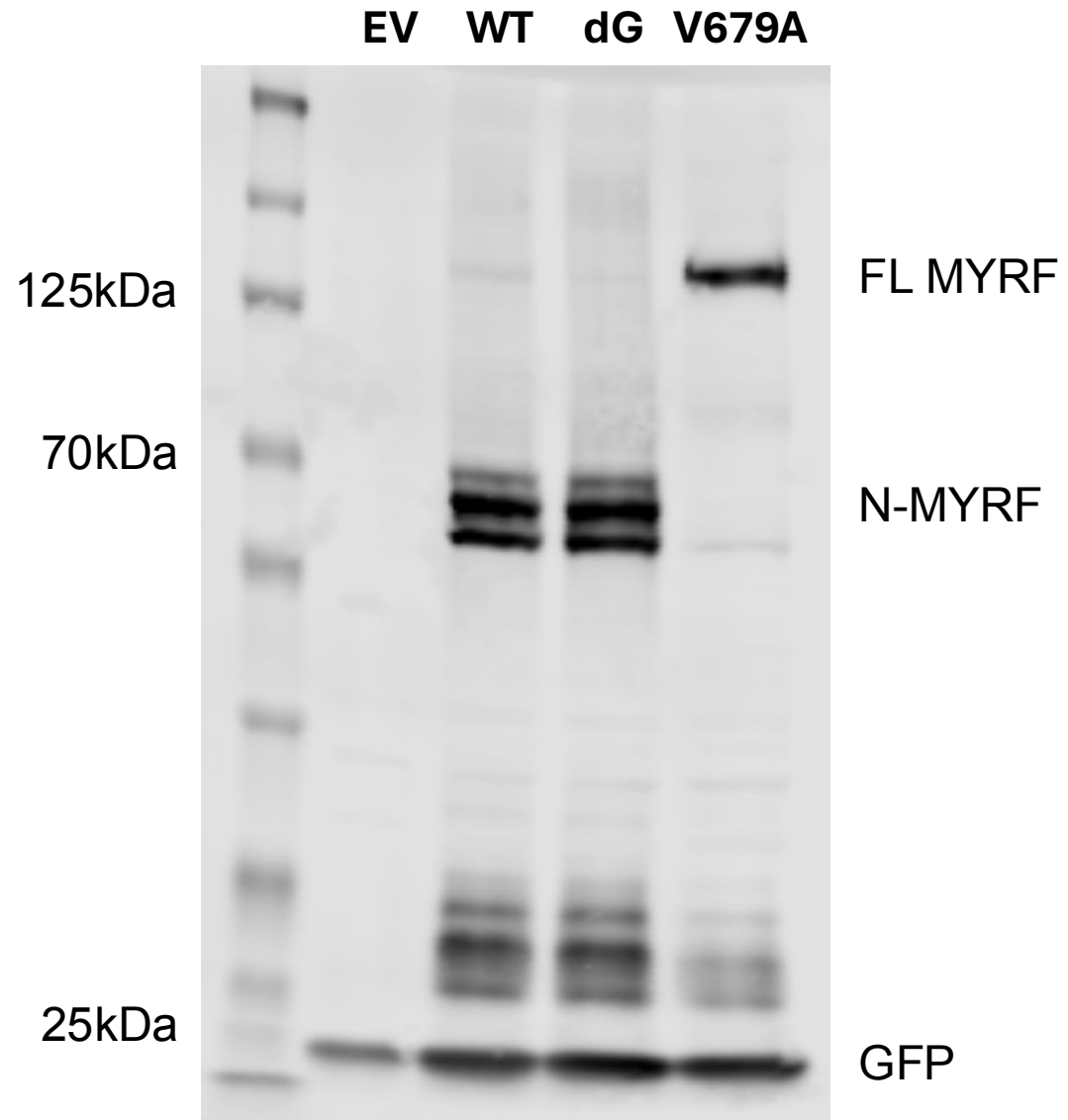

Full unedited blots for Supplemental Figure 1

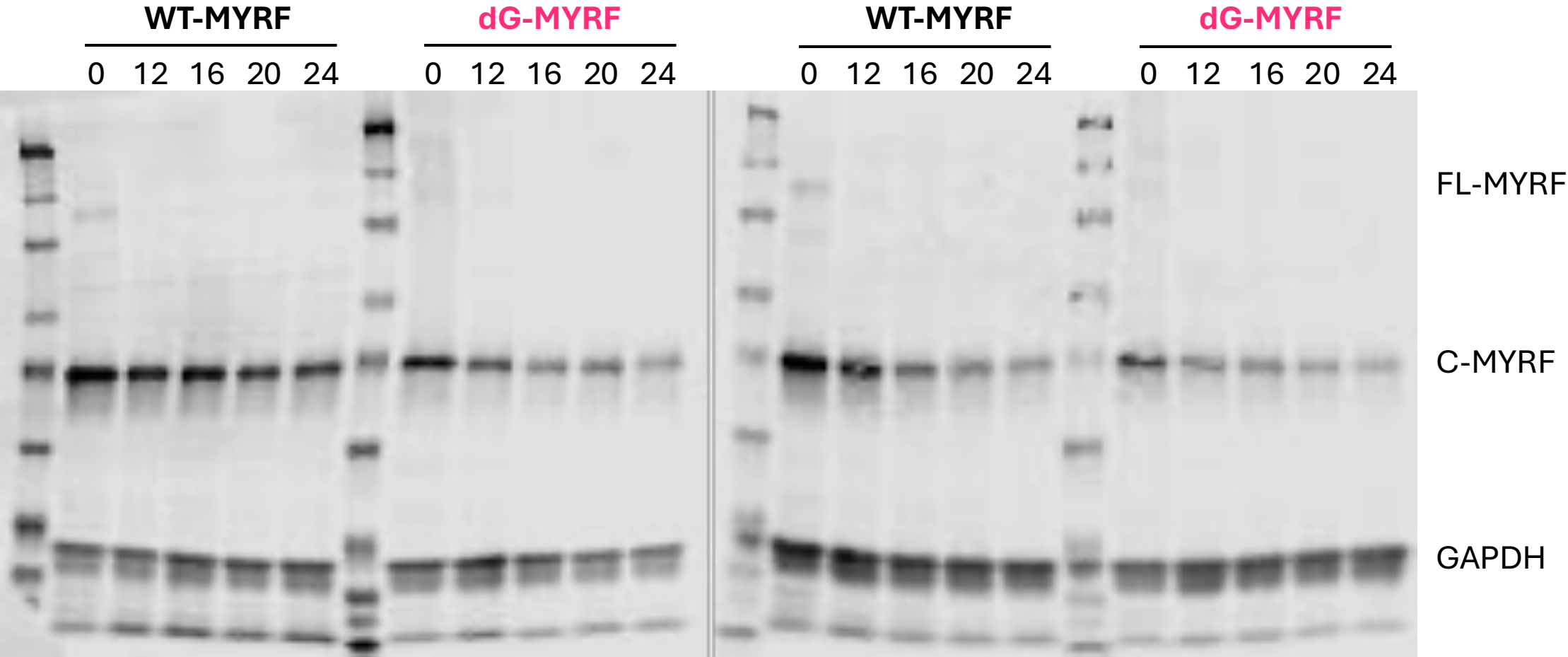

Supplement: Unedited blot and gel images [file jciinsight-11-194681-s213.pdf]
